# New Biobased Sulfonated Anionic Surfactants Based on the Esterification of Furoic Acid and Fatty Alcohols: A Green Solution for the Replacement of Oil Derivative Surfactants with Superior Proprieties

**DOI:** 10.1021/acssuschemeng.2c01766

**Published:** 2022-06-28

**Authors:** Amir Al Ghatta, Raul Contreras Aravenas, Yujie Wu, James Michael Perry, Jesus Lemus, Jason P. Hallett

**Affiliations:** Department of Chemical Engineering, Imperial College London, South Kensington Campus, London SW7 2AZ, U.K.

**Keywords:** biorenewables, surfactants, esterification, platform chemicals, sustainable chemistry

## Abstract

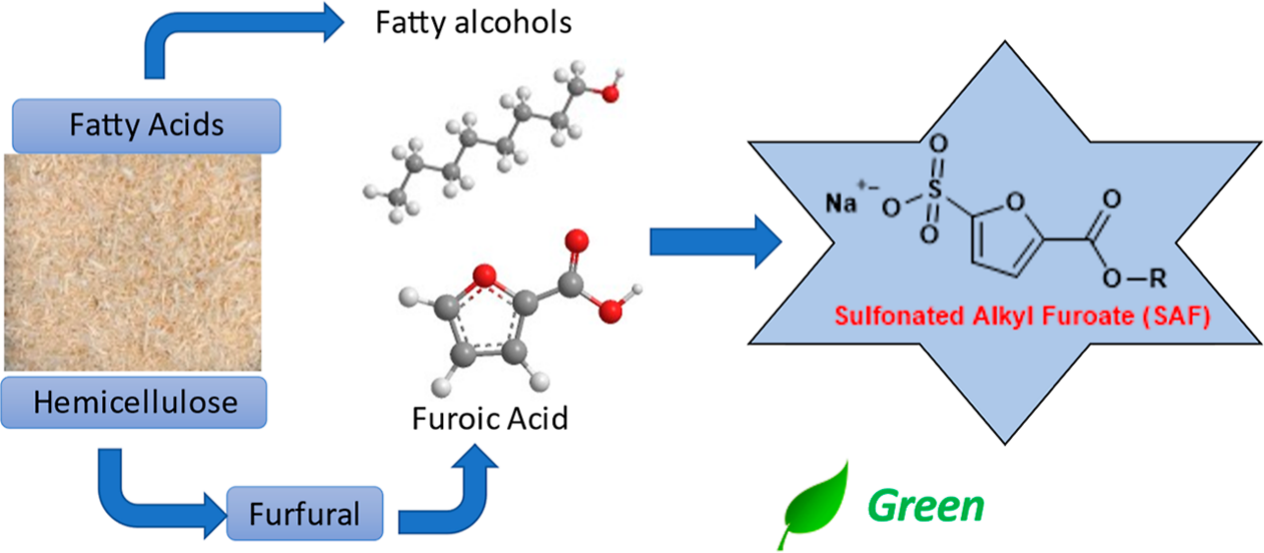

The surfactant market represents a key sector of the chemical industry and encompasses many diverse applications. Their sustainability in terms of feedstock used, synthetic procedure, biodegradability, and formulation are crucial parameters to assessing the environmental impact of the surfactant. The anionic surfactant linear alkyl benzene sulfonates have proven successful to date because of their high performance, low cost, and extensive studies within formulations to optimize performance, allowing usage in a large variety of applications, especially in cleaning. Due to their advantageous properties and extensive research and development, their substitution with a biobased surfactant such as sodium dodecyl sulfate has struggled to succeed. Furan surfactants have been reported as valuable candidates for the implementation of green alternatives to traditional anionic sulfonated surfactants with a perfect trade-off between performances and green credentials. However, their implementation suffers of scalability and high cost in producing the final product due to feedstock availability and low yields of the final product. Herein, we report a new class of furan surfactants, sulfonated alkyl furoates, which are derived from the esterification of furoic acid and fatty alcohols, followed by a sulfonation step. Compared to traditional surfactants, they showed more favorable behavior in basic proprieties (such as critical micelle concentration, ecotoxicity, hard water resistance, surface tension water/oil), which gives a good prospective for the introduction of a new biobased chemical with superior performances.

## Abstract 2

SAF is a new biobased surfactant that can replace oil-based analogues providing superior performances by exploiting fatty alcohol and furoic acid.

## Introduction

Surfactants are one of the most used commodity chemicals, commercialized in high volumes and finding applications in many different fields (household, industry, agriculture, personal care, oil and gas, food, and pharmaceuticals).^1^ The importance that these compounds have in different sectors makes them one of the most important synthetic chemicals produced globally. The market size of surfactants was 15 million tons/years in 2014 and is expected to continue expanding by 4.3% per year through 2022.^2^

An optimum surfactant design for a specific application is based on the careful selection of the chemical structure of the hydrophilic and hydrophobic parts as these structures strongly affect the surfactant’s properties. However, desirable surfactant properties differ according to the application. For example, it is desired that surfactants used for home care products have a low critical micelle concentration (CMC), a low Krafft temperature (KP), high solubility in water, high resistance to hard water, low toxicity, and high biodegradability.

Emulsifying properties are also important parameters for detergents and applications in enhanced oil recovery, while foaming and mildness are important factors for personal care. The increasing demand for more sustainable and greener products has added an extra requirement to the surfactant industry since product formulators are demanding biobased surfactants with equal performance to the current traditional petrochemical derivatives in the final application and remove all the petrochemical additives currently used to improve surfactant performance, which cause harm to the environment.^3−5^

Currently, the most used surfactants in industrial applications are anionic surfactants, and they account about 20% of a typical household product,^6^ particularly linear alkyl benzene sulfonates (LASs) and sodium dodecyl sulfate (SDS). LAS is a petroleum-based surfactant, while SDS is a biobased surfactant with several limitations such as low water solubility, low resistance to hard water, and low performances due to high CMC.^7^ A solution to improve detergent formulations in terms of sustainability and competitiveness is needed. This solution should be focused on synthesizing surfactants derived from low cost, biorenewable raw materials. These surfactants should also ideally exhibit high performance with a minimum use of additives. Consequently, researchers have sought biobased and biodegradable anionic surfactants that have performance and costs comparable to those of LAS.

Sugar derivatives have attracted much attention, and many catalytic routes have been developed to valorize sugars into furanic intermediates.^8,9^ Different paths have also been reported, which use furans to make surfactants.^10^ Lee and co-workers reported a 5-hydroxymethyl furfural (HMF)-derived sulfonated surfactant,^11^ but the product suffers from a complex and expensive synthetic route associated with HMF^12^ and low stability of the final product. Hoffman and co-workers^13^ have proposed a new class of biodegradable surfactants derived from photo-oxygenation of furfural with air, leading to a furanone head group. The author demonstrated the high biodegradability of these surfactants, but scalability of the process is limited by the photo-oxygenation step.

Recently, Dauenhauer and co-workers^7^ reported a new class of sulfonated furan alkyl surfactants based on the Friedel–Crafts acylation of furan. In their work, the authors evaluated the effect of substituting the benzene ring of LAS with a furan ring and analyzed different structural variants based on the hydrophobic chains. With the same alkyl chain, the new surfactant proved to have superior properties compared with the conventional LAS, including higher resistance to hard water. The authors demonstrated a strong correlation between the final proprieties of the surfactant with the furan linker and alkyl chain linker. A ketone linker proved to be detrimental for the final surfactant properties in terms of CMC, wettability, and resistance to hard water, requiring a reduction step of the ketone group to have good final surfactant properties. The performance of the furan alkyl surfactants after reduction proved to be higher than that of LAS, but major improvements are still required to decrease the Krafft point. Further studies for the efficient production of these surfactants were reported by implementing a high-temperature process by Vlachos and co-workers which used an iron-based catalyst to produce the ketone-based surfactant starting from furoic acid (FA) and fatty alcohol. However, the overall yield could not reach more than 50%.^14^ The group of Palkovits and co-workers^15^ has further reported another class of surfactants derived from condensation of furfuryl alcohol and fatty alcohol forming an ether linker, but this has proved to be unstable upon sulfonation, leading to complete degradation of the final product; therefore, the authors were not able to characterize the surfactant. Corma and co-workers proposed a highly efficient method to produce the carboxylate ether surfactant from HMF in a two-step process where the alcohol group is condensed with a fatty alcohol and the aldehyde group is oxidized into a carboxylic acid.^16^ However, the authors did not characterize its properties. Another group proposed an ester surfactant with the carboxylate head group derived from 2,5-furandicarboxylic acid (FDCA), proving good behavior of the surfactant toward biodegradation and aggregation properties.^17^

These efforts have demonstrated that the nature of the linker and the position of the sulfonate group strongly impact the properties of furan-based surfactants. Moreover, the choice of the furan source plays an important role on the feasibility of the process due to challenges in obtaining HMF and (biobased) furan at reasonable cost, which undermines the feasibility of the process at large scale.^18^

**Figure 1:**
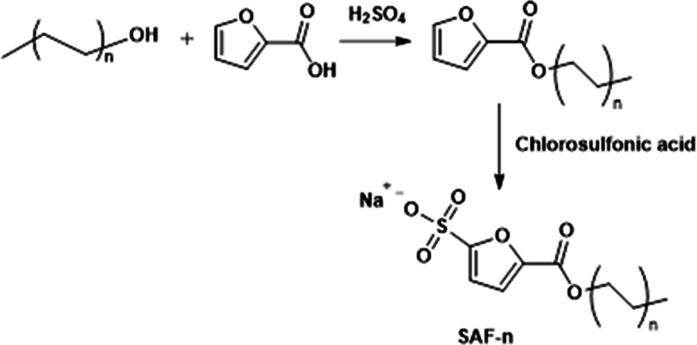
Reaction scheme used in this work for the synthesis of the new SAF.

In this work, we introduce a new highly scalable and biobased family of anionic surfactants called sulfonated alkyl furoates (SAFs, Figure 1) based on ester linkages. Compared with other reported furan surfactants, SAF unifies multiple advantages of furan properties by maintaining the high resistance to hard water with low CMC and high foamability with the further advantage of remarkably improving the Krafft point. Moreover, the synthesis route does not require the development of any specific catalyst or purification techniques which is usually reported for furan surfactants, making these products highly scalable. SAF also guarantees a more rational usage of fatty alcohols because of a more favorable atom economy, requiring about 50% lower fatty alcohol compared the equivalent SDS as the alkyl chain source. The introduction of the furan moiety through furfural and FA represents an exploitation of waste resources such as corn cob, putting all this within the context of the circular economy with the further advantage that these have the potential to be produced at lower cost compared with furan or HMF routes.^12,13,15^ While different linkers have been reported in the literature (ketone, C–C, ether), the ester linkage in combination with the sulfonated group has not been previously reported.

## Experimental Section

### Process Simulation by Aspen Plus

A property package was created in Aspen Properties v9.0., where the compounds used were defined as pseudo-components. The normal boiling point, density, and molecular weight were imported from COSMOtherm to create the pseudo-component. The COSMOSAC model was selected as the thermodynamic model, and a gamma method was modified to use COSMOSAC-Mathias modification. The σ-profile was specified as the pure component properties SGPRF1-5, and the COSMO volume was specified as the CSACVL component parameter.

Prices for technoeconomic assessment are reported in Table 1. Utility costs were used from one of our previous publications.^18^ Estimation of the capital costs was done using the integrated Aspen Economic package and the purchase and installation costs annualized over 10 years. Process simulations were done for a plant capacity for 2232 tons/year for alkyl furoate (AF) from furoic acid, 1948 tons/year for LAB evaluation, and 1948 tons/year for AF starting from furfural. The minimum selling price (MSP) and CO_2_ emissions were normalized per Kg of product.

**Table 1:** Prices for Evaluation of the Technoeconomic Assessment^a^

Furoic acid	4 $/Kg
Furfural	2 $/Kg
Dodecene	0.6 $/Kg
Benzene	0.83 $/Kg
Dodecanol	2 $/Kg
Steam cost for heat supply	0.12 $/Kg
Cooling water	0.056 $/m^3^
Waste water (primary treatment)	0.031 $/m^3^

a Production of AF from FA.

MSP was estimated as the sum of the feedstock, utility, and annualized capital costs. CO_2_ emissions were estimated as the methane required to generate steam at medium pressure (12 bar) with a thermal efficiency of 0.8. Heat integration was done respecting a pinch point of 5 °C. The energy required to reach the reaction temperature is considered supplied by external heating.

**Figure 2:**
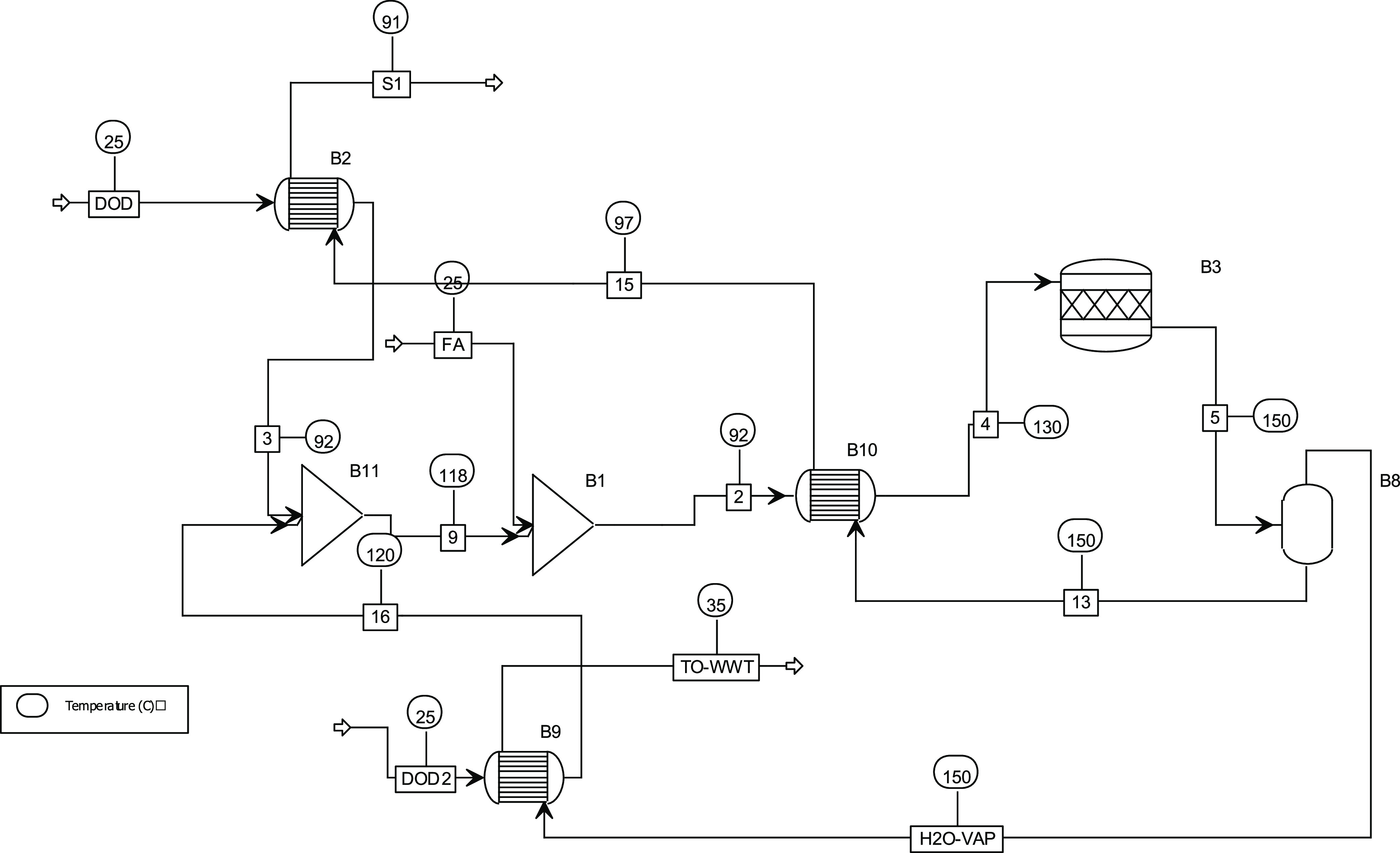
Process flowsheet of the production of AF starting from furoic acid FA and dodecanol (DOD).

Dodecyl furoate is produced according to the following process: two streams of dodecanol (DOD) were split to favor heat integration with the downstreams. After heat integration, FA is added at room temperature (B1) at a stoichiometric amount. The stream is preheated with the product stream and sent to the stoichiometric reactor (B3) at 150 °C. The water vapor generated is separated (B5) with a gas–liquid separator, condensed with heat recovery (B9), and sent to the wastewater treatment (WWT) system. The process is reported in Figure 2.

### Production of Alkyl Furoate from Furfural

**Figure 3:**
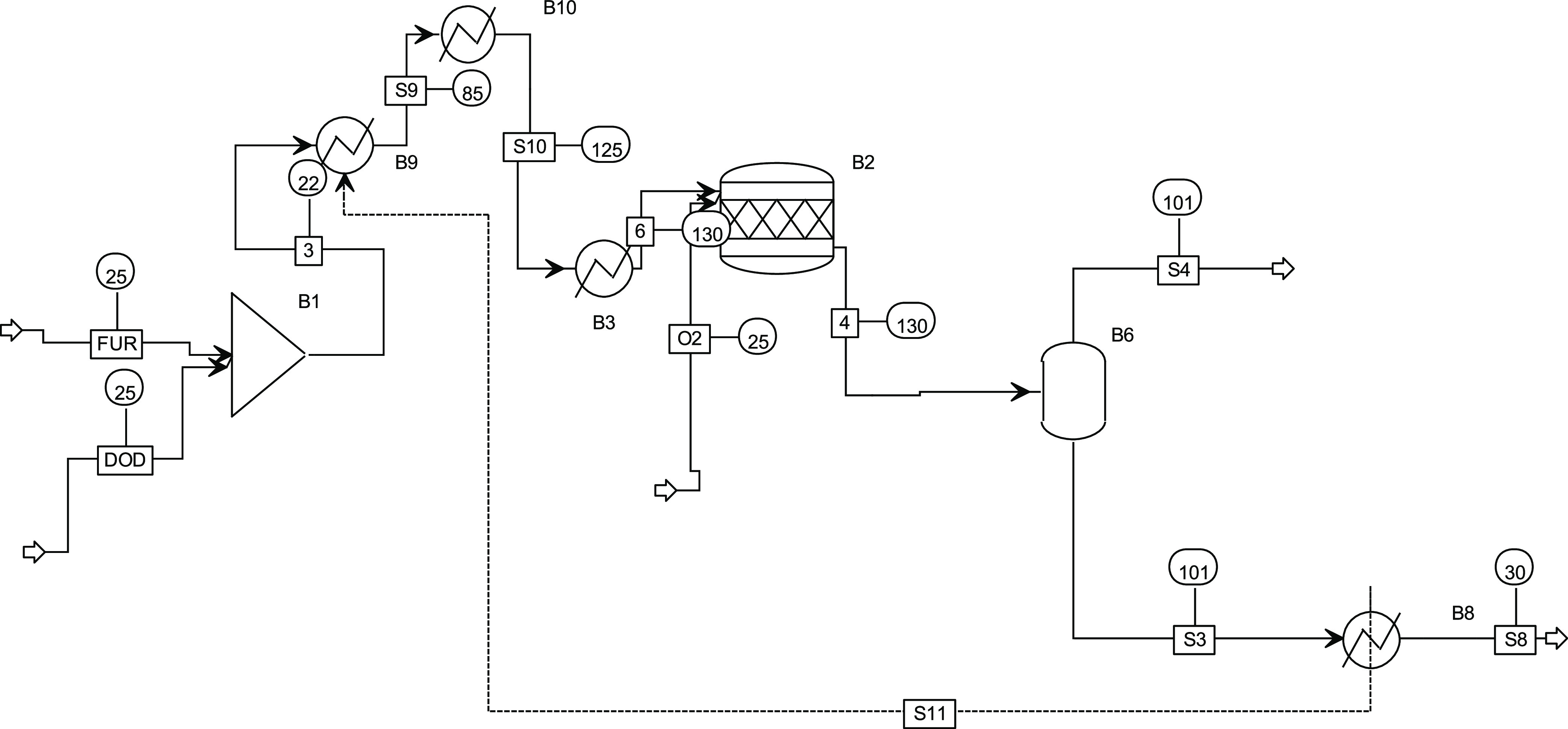
Process flowsheet of the production of the AF through oxidative esterification of furfural.

Furfural (FUR) and dodecanol are mixed at room temperature. The stream is preheated with the product stream (S3), then heated at 130 °C (B3) and reacted in B2 with pure oxygen (O_2_). The heat of reaction is removed with cooling water, and the cost is taken into account in the economics. The vapor is separated in B6, and the product is cooled at B8 with heat recovery. The process is reported in Figure 3.

### Production of LAB

**Figure 4:**
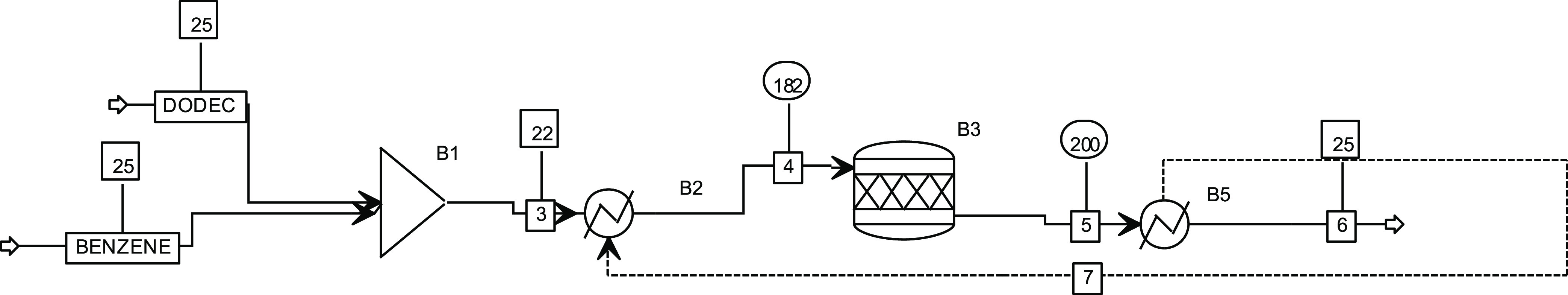
Process flowsheet to produce LAB from DODEC and benzene.

Dodecene (DODEC) and benzene are mixed at room temperature and preheated with heat recovery (B2). The reaction is carried out in reactor B3. The heat of reaction is removed with cooling water, and the cost is integrated in the economics. The process is reported in Figure 4.

### Catalyst Screening and Kinetics

Catalyst screening was conducted with the following procedure: a stoichiometric amount of furoic acid (FA) and dodecanol (DOD) (500 mg of FA, 830 mg of DOD) was placed in a 3 mL vial at room temperature. The catalyst was then added. In case sulfuric acid was used, 1 mol % was added, while in case Nafion, Amberlyst, and Purolite were used, 300 mg was added. The vial was placed in a preheated heating block at 150 °C for 1.5 h. During the reaction, the vials were vented through a needle to allow the removal of the water generated during the reaction.

For the kinetic experiments, 500 mg of FA was mixed with a stoichiometric amount of DOD (830 mg) and different amounts of sulfuric acid (0.41, 1, 1.5, 2%). The reaction mixture was placed in a preheated heating block at 150 °C for the desired time. This was repeated for different times. The mixture was analyzed by gas chromatography–mass spectrometry–flame ionization detection (GC–MS–FID) using naphthalene as the internal standard.

### Synthesis of the Furoate Esters

The reaction was performed with a Dean Stark apparatus to collect the water generated. FA (1 equiv) was mixed with a slight excess of DOD (1.05 equiv) and heated until the reaction mixture reaches a temperature of 150 °C. Then 1 mol % H_2_SO_4_ was added. The water was collected with a Dean Stark apparatus connected to a vacuum line with a pressure controller at 800 mbar. AF was obtained as a yellowish liquid. The product was confirmed by NMR and GC–MS. The residual FA content was assessed to be below 2% by HPLC.

#### Octyl Furoate

^1^H NMR (DMSO-*d*_6_): δ 8 (m, −C–CH–O−), 7.3, 6.7 (m × 2, 2 × 1H, 2 × C–CH–C), 4.23 (t, 2H, O–CH_2_–CH_2_, *J*_HH_ = 6.6 Hz), 1.66, 1.4–1.8 (m, CH_2_ alkyl chain), 0.86 (m, CH_2_–CH_3_). ^13^C{^1^H} NMR (DMSO-*d*_6_): δ 159.9 (C=O), 147.8 (CH–C–C=O), 144.6 (O–CH–CH), 119, 113 (−C–CH–CH–C), 65 (O–CH_2_–CH_2_), 31.7, 31.1, 29.1, 28.7, 25.8, 22.7 (6 × C, C–CH_2_–C), 14.4 (−CH_2_–CH_3_) ppm. MS (ES, −ve mode): *m*/*z* = 225.1486. FT-IR: 1705 cm^–1^ (O–C=O stretch).

#### Dodecyl Furoate

^1^H NMR (DMSO-*d*_6_): δ 7.9 (m, −C–CH–O−), 7.2, 6.7 (m × 2, 2 × 1H, 2 × C–CH–C), 4.23 (t, 2H, O–CH_2_–CH_2_, *J*_HH_ = 6.6 Hz), 1.66, 1.4–1.8 (m, CH_2_ alkyl chain), 0.86 (m, CH_2_–CH_3_). ^13^C{^1^H} NMR (DMSO-*d*_6_): δ 159.9 (C=O), 148.2 (CH–C–C=O), 144.5 (O–CH–CH), 119, 112.5 (−C–CH–CH–C), 65.7 (O–CH2–CH2), 31.8, 29.5, 29.2, 29.7, 28.8, 25.9, 22.6 (8 × C, C–CH2–C), 14.1 (−CH_2_–CH_3_) ppm. MS (ES, −ve mode): *m*/*z* = 281.2109. FT-IR: 1705 cm^–1^ (O–C=O stretch).

#### Hexadecyl Furoate

^1^H NMR (benzene-*d*_6_): δ 7.0, 6.9 (m × 2, 2 × 1H, 2 × C–CH–C), 5.8 (m, −C–CH–O−), 4.1 (t, 2H, O–CH_2_–CH2, *J*_HH_ = 6.7 Hz), 1.44, 1.37–1.05 (m, CH_2_ alkyl chain), 0.85 (m, CH_2_–CH_3_). ^13^C{^1^H} NMR (benzene-d6): δ 158.5 (C=O), 145.7 (CH–C–C=O), 145.5 (O–CH–CH), 117, 111 (−C–CH–CH–C), 64.4 (O–CH2–CH2), 32, 29.9–29.3, 28.8, 26, 23 (6 × C, C–CH2–C), 14.1 (−CH_2_–CH_3_) ppm. MS (ES, −ve mode): *m*/*z* = 337.2735. FT-IR: 1705 cm^–1^ (O–C=O stretch).

### Synthesis of SAF

The furoate ester (10 g) was added to dry chloroform (5 mL) and mixed at room temperature until full dissolution was achieved. Chloroform was needed to avoid foaming, which for large batches represented an operative problem. The reaction flask was then connected to a water-filled bubbler through which the produced HCl was vented. Chlorosulfonic acid (CSA) was then added (1.05 equiv). The water was first saturated by HCl and then bubbling began, indicating consumption of CSA. The reaction was left until no further bubbling was observed, and the mixture analyzed by ^1^H NMR spectroscopy to confirm that the reaction was complete with full conversion of the ester and consumption of most of the CSA (CSA shift: 10.9 ppm). Chloroform was removed under vacuum to afford a dark-green solution, which was diluted with water and neutralized to pH 7. The mixture was then placed in a fridge, where most of the surfactant precipitated as a white solid. The product was collected by precipitation and washed with cold water. Analysis by ion chromatography confirmed that residual sulfate and chloride salts are below 0.2%.

#### Sulfonated Octyl Furoate

^1^H NMR (D_2_O): 6.9, 6.7 (m × 2, 2 × 1H, 2 × C–CH–C), 4.2 (t, 2H, O–CH_2_–CH_2_, *J*_HH_ = 6.6 Hz), 1.6, 1.34–1.0 (m, CH_2_ alkyl chain), 0.8 (m, CH_2_–CH_3_). ^13^C{^1^H} NMR (D_2_O): δ 158.5 (C=O), 155.8(O–CH–CH) 144.5 (CH–C–C=O), 118.2–112.2 (−C–CH–CH–C), 66.1 (O–CH_2_–CH_2_), 31.7, 29.2, 29.1, 28.3, 25.7, 22.6 (6 × C, C–CH_2_–C), 13.8 (−CH_2_–CH_3_) ppm. MS (ES, −ve mode): *m*/*z* (abundance) = 303.087 (C_13_H_19_O_6_S^–^ 100%). FT-IR: 1250–1300 cm^–1^, (S=O stretching), 1705 cm^–1^ (O–C=O stretching).

#### Sulfonated Dodecyl Furoate

^1^H NMR (DMSO-*d*_6_): δ 7.9 (m, −C–CH–O−), 7.2, 6.7 (m × 2, 2 × 1H, 2 × C–CH–C), 4.23 (t, 2H, O–CH_2_–CH_2_, *J*_HH_ = 6.6 Hz), 1.66, 1.4–1.8 (m, CH_2_ alkyl chain), 0.86 (m, CH_2_–CH_3_). ^13^C{^1^H} NMR (DMSO-*d*_6_): δ 159.9 (C=O), 148.2 (CH–C–C=O), 144.5 (O–CH–CH), 119, 112.5 (−C–CH–CH–C), 65.7 (O–CH_2_–CH_2_), 31.8, 29.5, 29.2, 29.7, 28.8, 25.9, 22.6 (8 × C, C–CH_2_–C), 14.1 (−CH_2_–CH_3_) ppm. MS (ES, −ve mode): *m*/*z* (abundance) = 359.0865 (C_17_H_27_O_6_S^–^ 100%). FT-IR: 1100–1300 cm^–1^, (S=O stretching), 1705 cm^–1^ (O–C=O stretching).

#### Sulfonated Hexadecyl Furoate

^1^H NMR (D_2_O): δ 6.9, 6.7 (m × 2, 2 × 1H, 2 × C–CH–C), 4.1 (t, 2H, O–CH_2_–CH_2_, *J*_HH_ = 6.4 Hz), 1.54, 1.26–0.95 (m, CH_2_ alkyl chain), 0.8 (m, CH_2_–CH_3_). ^13^C{^1^H} NMR (D_2_O): δ 159.5 (C=O), 156.5(O–CH–CH) 144.6 (CH–C–C=O), 118.9–112.9 (−C–CH–CH–C), 66.1 (O–CH_2_–CH_2_), 32.2, 31–29.3, 28.5, 26, 22.8 (10 × C, C–CH_2_–C), 13.8 (−CH_2_–CH_3_) ppm. MS (ES, −ve mode): *m*/*z* (abundance) = 415.1465 (C_21_H_35_O_6_S^–^ 100%). FT-IR: 1100–1300 cm^–1^, (S=O stretching), 1705 cm^–1^ (O–C=O stretching).

## Results and Discussions

### Synthesis and Sustainability of SAF

The usage of furan building blocks has attracted much interest in the last 20 years for the production of different biobased chemicals which can substitute bulk petrochemicals. For example, different catalytic pathways using HMF, furfural, or furan have been used;^7−9,11−13^ however, a technoeconomic analysis which estimates the environmental impact and price viability was never reported for these compounds, even though purification techniques and feedstock price are crucial parameters to define the success that these surfactants can have in the market.

In order to establish a sustainable process, the synthesis methodology is extremely important since it directly impacts the CO_2_ emissions, energy requirements, and waste generation.^18^ The development of an efficient catalytic pathway should be focused on high yields at short reaction times, minimizing the requirements of purification steps (such as distillation or solvent washing) to avoid high operating costs which would compromise the feasibility of the process.

**Figure 5:**
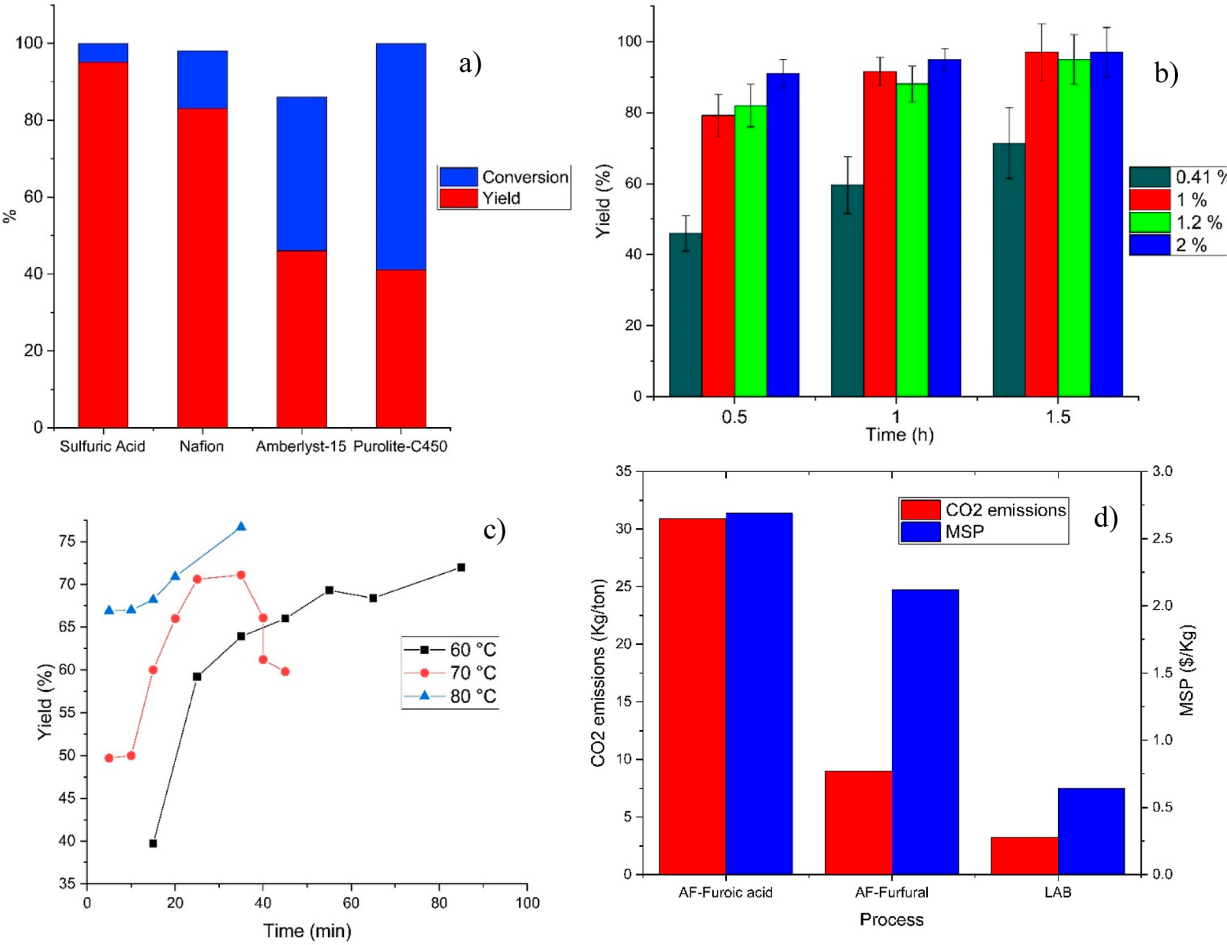
Evaluation of the catalytic synthesis of SAF: (a) catalyst screening at 150 °C, 1.5 h, a stoichiometric amount of DOD/FA, 1% H_2_SO_4_, 300 mg of Nafion, Amberlyst-15, and Purolite; (b) kinetic analysis of the esterification at different mol % ratios, 1:1 reactant ratio, 150 °C; (c) kinetics of the sulfonation of octyl furoate with CSA at the stoichiometric ratio at different temperatures; (d) evaluation of the CO_2_ emissions and MSP of the unsulfonated surfactant starting from FA and furfural in comparison with LAB.

In our first analysis in performing the first step of the reaction scheme reported in Figure 1, it was observed that the reaction between FA and DOD is autocatalytic when these are contacted at 150 °C, reaching up to 60% of yield in 6 h, and that a venting of the reaction system was needed to allow the water to evaporate and allow the equilibrium to be pushed toward the products. Under catalyst-free conditions, the reaction rate decreased remarkably after 6 h, probably due to low reactant concentrations arising from high conversion. For this reason, a catalyst addition was evaluated to push the conversion further. Different Bronsted acids were evaluated in homogeneous and heterogeneous forms in order to increase the yield (Figure 5a). Sulfuric acid and Nafion proved to be catalysts which gave a high yield (over 95%) at a short reaction time (1.5 h) and provide two valid options for homogeneous or heterogeneous catalysis according to the specification of the final product. If homogeneous catalysis is adopted, the removal of sulfuric acid can be achieved by washing with water or distillation; however, both these can lead to excessive waste generation or energy usage, which could undermine the green credentials of the process and increase the operating cost. In this case, the final product will need to undergo the sulfonation step with residual acid which will result in a higher salt content in the sulfonated product. This can be acceptable depending on the final application at which the surfactant is aimed (such as detergents) since residual salts can affect the mildness and solubility of other components in the final formulations. Moreover, a high sulfuric acid content can lead to corrosion of equipment for an eventual scale-up; therefore, minimization of the catalyst content is an essential parameter for the optimization of the technology. From the optimization study reported in Figure 5b, it could be deduced that the sulfuric acid content can be decreased down to 1% without excessively affecting the reaction kinetics.

Figure 5c presents the results of the solvent-free sulfonation performed with CSA (second step of Figure 1) at different temperatures at a 1:1 M ratio. At temperatures above 70 °C, the sulfonic acid undergoes rapid degradation at long reaction times. An increase in yield at 80 °C was observed; however, the mixture turned into a dark solid which strongly compromises the quality of the final product concerning color specification. Indeed, even in the attempt of washing with the final surfactant with different organic solvents, the final product struggles to achieve a clear color. Sulfonation at low temperature leads to an optimum sulfonation yield of 72%, avoiding excessive darkening of the solution. However, the sulfonation using liquid sulfonating agents (such as CSA and oleum) is not used anymore in the chemical industry for commodity surfactants due to the low control of the quality of the final product. Further optimization on the color and yield can be achieved by utilizing SO_3_ as a milder sulfonating agent in a falling film reactor which today is used at large scale to minimize the cost and improve the quality of the final product, in addition to a better atom economy which is equivalent to 100% for the SO_3_ process and 91% for the CSA option due to the formation of HCl as a side product. The main advantage of the falling film reactor is that SO_3_ can be stripped out the reaction mixture and neutralization can be performed quicker, reducing the side products that can be formed under acidic conditions.^19^

We also conducted an evaluation of the MSP and CO_2_ emissions in producing AF (Figure 5d) and compared these with the alkylation of benzene with dodecene (DODEC) to produce linear alkyl benzene (LAB). The analysis refers to the production of the intermediate unsulfonated product since the large-scale sulfonation step is similar for each product. The production of the SAF intermediate (AF) was evaluated through two potential routes: esterification of fatty alcohol with FA and oxidative esterification of furfural. Detailed results on the CAPEX and OPEX are reported in the Supporting Information (Table S3).

The process which uses FA resulted in higher CO_2_ emissions and a higher MSP since energy is required to remove the water through evaporation with the further disadvantage of low heat of integration due to low heat of reaction. Remarkable advantages can be obtained using furfural as feedstock due to its lower price and the exothermic nature of the oxidation which provides heat to satisfy the energy demand of the plant, making the process more economically competitive. Moreover, the process starting from furfural has a similar atom economy (93.2%) compared to the process using FA (94%). The process modeled for producing LAB yields a more competitive price, mainly due to the low price of the feedstock which has a well-established supply chain in the commodity chemical market. However, further considerations exist concerning the sustainability of the usage of petroleum-derived feedstocks since they need to undergo multiple transformations starting from paraffins and BTX (benzene, toluene, and xylene) which are associated with high greenhouse gas emissions. Moreover, end of life emission of LAS is the main contributor to the total emissions and estimated to be 2.3 kg_CO2_/kg_LAS_.^20^ While the CO_2_ emissions estimated for the production of SAF are low, the life cycle of furfural and FA are crucial to define the overall environmental impact of SAF. However, currently, the supply chain for these feedstocks is not as fully developed as for petroleum-based chemicals, and an exemplary, optimized process at large scale is still not constructed.

### Evaluation of Surfactant Properties

**Figure 6:**
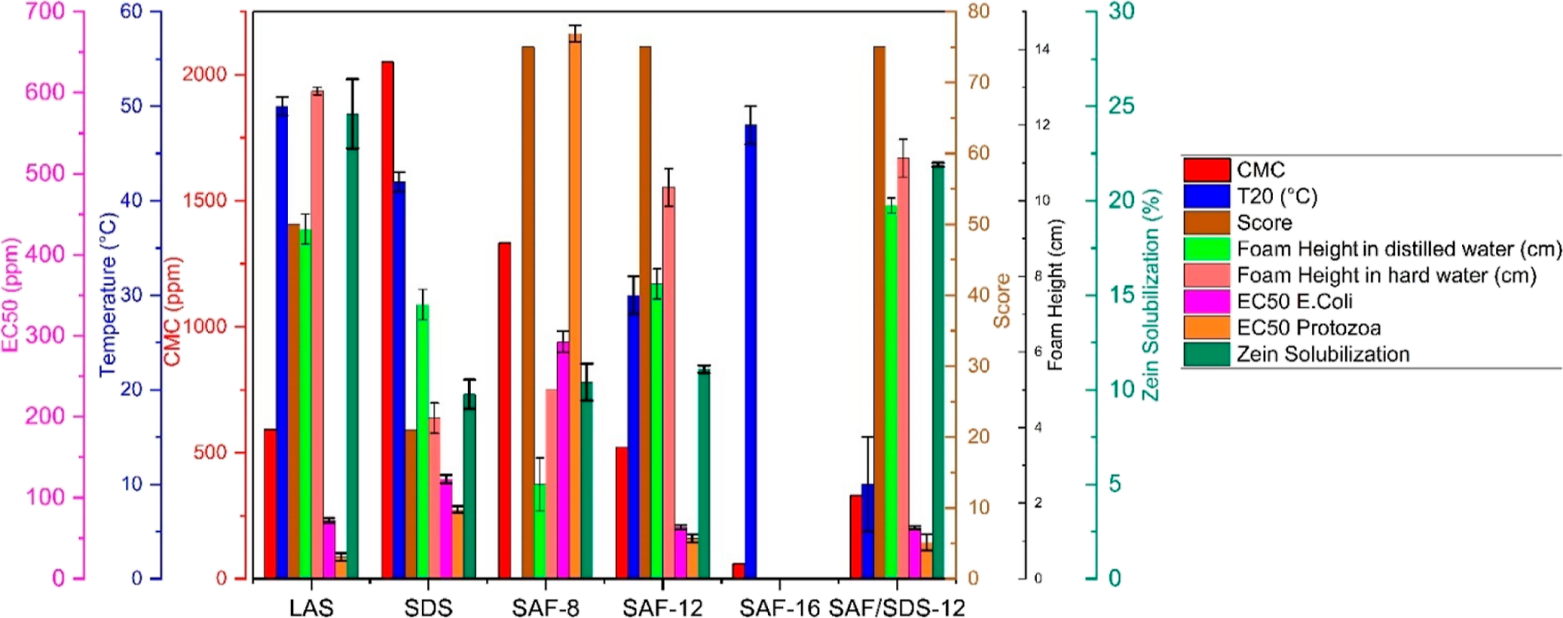
Property evaluation of different surfactants: CMC, temperature to solubilize 20% of the surfactant in water (*T*_20_; c), resistance to hard water according the ISO score methodology, foamability of the surfactant expressed as the height of the foam after 5 min, concentration to inhibit bacterial growth by 50% (EC50), and Zein solubilization at 0.5% surfactant concentration at 40° for 12 h. SAF/SDS evaluated in a mass ratio of 6:1.

The properties of the surfactant were evaluated under salt-free conditions (<100 ppm) according to the procedure reported in the methodology. The results reported in Figure 6 demonstrate that SAF displays some unique behavior compared with traditional anionic surfactants (LAS and SDS). Of particular note, SAF has favorable CMC and solubility in water (Tables S4 and S6), which suggests the potential for superior detergency as low CMC is desirable in cleaning formulations. SAF-8 exhibits a lower CMC than commercial SDS, while SAF-12 and SAF-16 have a lower CMC than LAS, indicating that SAF could function as a replacement for traditional surfactants in a variety of home and personal care products. While the CMC is a good estimation for micelle formation, it remains a rough estimation for the performance that the surfactant can have in specific applications such as in oil recovery or detergency. A more rigorous estimation comes from measuring the decrease in surface tension between water and hydrophobic solvents. A lower surface tension corresponds to a more favorable cleaning ability since it favors the formation of stable emulsions. The droplet formation of a water/surfactant solution in octanol has been reported for SAF-8, SAF-12, and LAS (Table S7) at a surfactant concentration of twice the CMC. SAF-8 shows the lowest surface tension (2.65 ± 0.32 mN/cm) due mostly to the high concentration of the surfactant used (4298 ppm). SAF-12 shows a surface tension (3.42 ± 0.58 mN/cm) lower compared to that of LAS (4.48 ± 0.75 mN/cm), further indicating the potential for a high cleaning capability and emulsifying properties of this new category of sulfonated surfactants.

SAF-8 proved to be remarkably soluble in water, reaching saturation at a temperature below 0 °C (Figure 6). We observed that at room temperature, the solubility of this surfactant exceeds 80% w/w, which represents a unique characteristic for this type of aromatic surfactant. However, a gradual increase in *T*_20_ is observed with longer alkyl chains. The solubility showed a strong dependence with temperature, with the saturation of SAF-12 increasing from 2 to 28% on increasing the temperature from 25 to 30 °C. The same behavior was observed for SAF-16 at a higher temperature (47 °C). Attempts to stabilize SAF-12 at lower temperatures with the extensively used hydrotrope *p*-xylene sulfonic acid failed. However, we observed that the solubility increases remarkably by using SDS as a cosurfactant at a mass ratio of 6:1 (SAF-12/SDS). This allowed us to stabilize SAF at a lower temperature, reaching a solubility of total surfactant concentration over 30% without the addition of any hydrotrope. It is worth mentioning that this mixture exhibits a lower CMC than SDS or SAF-12 alone, which is an indication of synergy between the two surfactants.

SAF 8 and SAF-12 exhibit high resistance to hard water, which is a noteworthy weakness for both LAS and SDS. This aspect was analyzed in a previous study by Dauenhauer and co-workers,^7^ who showed that the linker between the aromatic head group and the aliphatic chain has a strong influence on the surfactant precipitation in hard water. In that study, a functionalized linker such as a ketone significantly decreased the solubility of the surfactant in hard water, compared with C–C linkers. The fact that the ester linker utilized here proved beneficial for solubility in hard water eliminates the need to perform a hydrogenation step to defunctionalize the linkage, representing a further advantage of SAF in terms of scalability. The mixture of SAF-12/SDS also proved to be highly stable in hard water, further confirming a synergetic effect of the mixture of these two compounds in optimizing the CMC, solubility, and hard water resistance.

Further studies were performed to determine the foamability of the surfactant in distilled and hard water (Table S8), which is an important parameter for personal care formulations. LAS shows the highest foamability, but its use in the personal care sector is strongly limited because of its very low mildness and petrochemical origin. This is the reason why many current personal care products use SDS instead of LAS. SDS shows very good foamability in distilled water, but this decreases significantly in hard water due to high instability, which leads to precipitation. The stability of the foam is improved using SAF surfactants; specifically, SAF-12 shows higher foamability compared with SDS, which is further improved in hard water, making this compound very suitable as a new biobased surfactant which does not require any chelants or a foam boaster, which SDS does require.

The applicability of SAF in personal care applications was further studied by analyzing the mildness of the surfactant through the Zein solubilization test (Table S10). This test assesses skin irritation through the solubilization of the Zein protein through denaturation when in contact with the surfactant. We used a methodology similar to that reported by Cohen and co-workers.^21,22^ Surprisingly, SAF-8 and SAF-12 showed better mildness values compared with the commercial LAS and SDS, suggesting a less irritating effect for those applications which involve skin contact.

The superior properties of SAF can greatly simplify the design of formulations for different applications in the consumer sector. Specifically, the combination SAF-12/SDS has the potential to exhibit high cleaning ability due its low CMC and the low surface tension of water–oil which is provided by SAF-12. In order to further analyze the sustainability of SAF, ecotoxicity was evaluated through the inhibition of *E. coli* in expressing b-galactosidase and inhibition of growth of protozoa microorganisms, both quantified by EC_50_ (Table S9). Our results indicate that the alkyl chain has a much higher influence compared with the head group on the toxicity. SAF-8 proved to have a much lower toxicity compared with the 12-carbon chain surfactants. SAF-12 proved to have a higher toxicity than SDS, possibly due to the presence of the ester group. However, SAF-12 exhibited lower toxicity compared to LAS with both methodologies, indicating a more beneficial effect of the furan ring compared with the aryl ring, further establishing SAF-12 as an eco-friendly alternative to LAS.

## Discussion

Our studies on SAF have demonstrated that this surfactant can exhibit superior properties compared with previously reported furan-based surfactants, further demonstrating the importance of the linkage between the alkyl chain and the furan ring. The properties reported by Dauenhauer and co-workers were retained while using an ester linkage instead of a C–C chain (2). The usage of ester linkage makes the scale-up of the alkylation viable since it can be carried out in one step without the need of a purification step; however, the usage is limited to those applications where pH is neutral due to risk of hydrolysis of the ester group. SAF’s properties are superior compared with the ketone linker (3), which proved to be unsatisfactory in terms of CMC and resistance to hard water and proved to be stable under light. The carboxylate surfactant has been reported in the literature with ether (5), (6) and ester linkers, but the synthesis route reported requires HMF or FDCA, which reduces the economic viability of these surfactants. Moreover, carboxylate surfactants are known to exhibit poor resistance to hard water, which limits their performance. In Table 2, we report different properties which compare SAF with other surfactants reported in the literature.

**Table 2:**
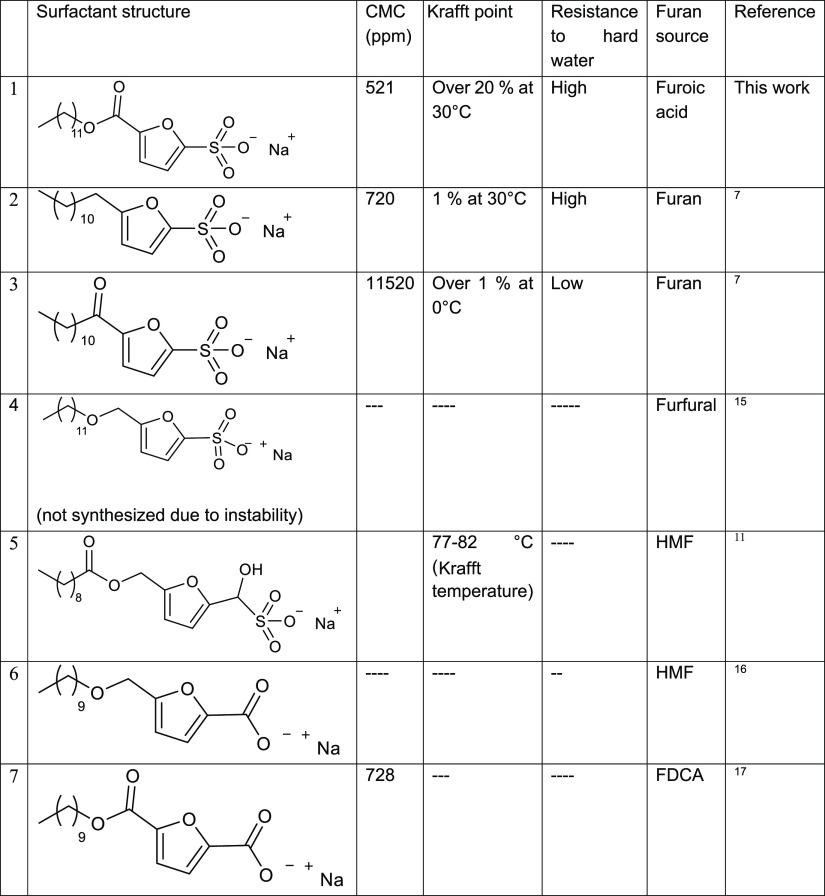
Comparison of Different Furan-Based Surfactant Properties Reported in the Literature

Further improvements of the overall performance can be achieved by mixing SAF with SDS with improvements in all properties, retaining high resistance to hard water, an area where SDS is weak.

The synthesis procedure of SAF has multiple advantages over previously reported furan surfactants since the intermediate can be produced at high purity and yield without the need of any purification step, with the further advantages of a reduction in the reaction steps and improved secondary properties (color), which are important for obtaining a final surfactant with good specification.

FA can be produced from furfural with multiple reported oxidation techniques which already are efficient and provide high yield. Currently, this product is not commercialized at large scale since there are no applications in commodity chemicals and further studies are needed to implement these processes. The implementation of the production of AF directly from furfural through oxidative esterification can lead to further improvements of the process economics, but a suitable catalyst needs to be developed to perform this reaction under stoichiometric conditions which can avoid the implementation of a distillation column to remove the fatty alcohol, which would increase the cost.

The implementation of an efficient process for the production of FA has the potential to establish SAF as a new economically competitive commodity chemical with higher performance and green credentials compared with traditional surfactants and potential in emulsification applications such as cleaning and oil recovery.

## Conclusions

In this study, we explored SAF as a potential replacement of LAS in formulation development. The advantages in using SAF lie on the efficient formation of the hydrophobic part of the molecule at high yield simply using sulfuric acid as a catalyst and without the requirement of a purification step, reducing the energy requirements of the plant. This can significantly reduce the operating costs of a scale-up which can guarantee an acceptable MSP in association with low CO_2_ emissions related to the processing, which gives the prospective of a competitive market price. Ultimately, the low-cost production of FA and the compatibility of the sulfonation step in the falling film reactor will define the success of this technology.

The basic proprieties of SAF suggest that it can guarantee similar or better performances than LAS in terms of emulsification proprieties, detergency, and personal care thanks to the low surface tension of water–oil, CMC, and skin irritation. Moreover, the high resistance in hard water and the improvement of CMC and solubility when SAF is mixed with SDS open the prospective of a new cheaper formulation design with high performances.

Preliminary results on the ecotoxicity suggest that this surfactant does not exhibit any environmental concern compared to LAS. Further studies need to be addressed on the anaerobic and aerobic biodegradability of this surfactant.

## Supporting Information Available

Experimental section and numerical results regarding; technoeconomic assesment; solubility; resistance to hard water; CMC and surface tension measurements; foaming tests; ecotoxicity tests; Zein test; NMR spectra; mass spectra; FT-IR (PDF)
