# Supplementary material for: New Biobased Sulfonated Anionic Surfactants Based on the Esterification of Furoic Acid and Fatty Alcohols: A Green Solution for the Replacement of Oil Derivative Surfactants with Superior Proprieties
Source: ACS Sustain Chem Eng. 2022 Jun 28;10(27):8846–55. doi: 10.1021/acssuschemeng.2c01766 (PMC9278057; doi:10.1021/acssuschemeng.2c01766)
Supplement: Supplementary file 1 — sc2c01766_si_001.pdf [file sc2c01766_si_001.pdf]

Supporting information

New biobased sulfonated anionic surfactants based on the esterification of furoic acid and fatty alcohols. A green solution for the replacement of oil derivative surfactants with superior proprieties

Amir Al Ghatta*, Raul Contreras Aravena, Yujie Wu, James Michael Perry, Jesus Lemus and Jason P. Hallett*

Department of Chemical Engineering, Imperial College, South Kensington Campus, London SW7 2AZ.

Department of Chemistry, Imperial College, Molecular Sciences Research Hub, White City Campus, London W12 0BZ.

e-mail: a.al-ghatta16@imperial.ac.uk, j.hallett@imperial.ac.uk

Contents

Experimental Section 3

Materials 3

Quantum Chemical Calculations by TURBOMOLE and COSMO-RS 3

Solubility 3

Resistance to hard water 3

Critical Micelle Concentration (CMC) 4

Surface tension measurements 4

Foaming tests 5

Inhibition of secretion B-galactosidase from E.Coli 5

Inhibition of Protozoa growth 6

Zein Test 6

Results and Discussion 6

Technoeconomic assessment 6

Solubility study 7

Resistance to hard water 7

Critical micelle concentration and surface tension water-octanol 7

Foam Test 8

Evaluation Ecotoxicity 9
Zein Test 9
NMR Spectra 9
Mass spectra 15
FT-IR 16
CMC Measurement 19
Surface tension measurement 19
References 19

Number of pages : 19

Number of figures: 24

Number of tables: 10

Experimental Section

Materials

Furoic Acid (98 %), 1-octanol (>99 %), 1-dodecanol, 1-hexadecanol (99 %), chlorosulfonic acid (99%), pharmaceutical grade sodium dodecylbenzenesulfonate (LAS) and sodium dodecyl sulfate were obtained from Sigma Aldrich. Purolite-C450 was supplied by Purolite. Dichloromethane (DCM) and chloroform were obtained from VWR.

Chloroform was purified from ethanol by washing with water (3 times 3:1 ratio) and distilled over phosphorous pentoxide in a distillation tower. The solvent was stored in a dark container to minimize decomposition. All other chemicals were used without purification unless otherwise stated.

Quantum Chemical Calculations by TURBOMOLE and COSMO-RS

The chemical structure of the compounds used (furfural, furoic acid, dodecanol dodecene, benzene, alkyl furoate and dodecyl benzene) were created and optimized using TURBOMOLE v7.02 (with TmoleX v4.2.1 graphic interface) software. Quantum chemical calculations were carried out to generate a COSMO file containing energies, geometries, and the polarization charge of the σ-surface. The standard method used in COSMOtherm is a B88-P86 (bp) functional (32) and a TZVP basis set with RI approximation (33) using the COSMO solvation model. The COSMO-RS method program package (version C30_1801) and its parametrization BP_TZVP_C30_18 were used in COSMOtherm software. In this work, the COSMO-RS method was used to create pseudo-components into Aspen Properties. COSMOtherm was used to carry out the normal boiling point, density, σ-profile, and COSMO-volume calculations (the last two to use the COSMOSAC model from Aspen Properties).

Solubility

Solubility was evaluated as the temperature to solubilize 20 % of surfactant. In brief 200 mg of surfactant were placed in a glass vial and 1 ml of water was added. The vial was placed in a pre-heated heating block at a certain temperature and gradually heated (2 °C) and left for 1 h under stirring. The procedure was repeated until full solubilization was achieved.

Resistance to hard water

These measurements were performed according the protocol reported in ISO standard 1063 with some minor modifications. Calcium chloride was used as the cation source. In brief, three different solutions of a Ca salt at 6, 9 and 12 mq/L (3.0, 4.5,6.0 mM) were prepared. A mother solution of surfactant with 50 mg/mL was prepared in water at 20 °C. A defined aliquot of the mother solution was transferred into a 50 mL Falcon tube and diluted to 50 mL with the calcium solution to be tested. Five different aliquots of surfactant mother solution were tested: 5.0, 2.5, 1.2, 0.6 and 0.3 mL. For every test, the solution was assigned a score according to Table S1. The scores of the 15 tests for each surfactant were summed and the final result was expressed as the mean stability in Table S2.

Table S1 Score corresponding to the appearance of the liquid according to ISO standard 1063. A liquid which is not clear, but through which objects can be seen, is regarded as opalescent. A liquid which is not clear, but through which objects cannot be seen, is regarded as cloudy.

Appearance of the liquid	Score number
Clear	5
Opalescent	4
Cloudy	3
Slight precipitate	2
Heavy precipitate	1

Table S2 Mean stability table according to ISO standard 1063.

Total Score	Mean Stability
15-18	1
19-37	2
38-56	3
57-74	4
75	5

Critical Micelle Concentration (CMC)

The surface tension of water-surfactant solutions was evaluated at concentrations between 10 ppm and 12.5 ppm. The specific concentrations evaluated for each surfactant varied depending on the solubility of the surfactant and the surface tension reduction observed during the analysis. The experiments were performed at 25 °C. Deionized water was used for each experiment.

The pendant drop method was used to calculate the surface tension by using a Krüss DSA25. A drop of the solution was produced in the tip of a blunt metal needle (1.25 mm) until the drop reached its maximum size. After 10-15 seconds of equilibration, an image of the drop was captured using a digital camera. The drop was analysed using the software Krüss EasyDrop Standard Drop Shape Analysis (DSA1) v 1.92. The software uses the Young-Laplace equation to calculate the surface tension according to the drop deformation considering the effects of gravity and hydrostatic pressure.

Critical micelle concentrations (CMC) were obtained after plotting surface tension vs surfactant concentration. The CMC is calculated as the surfactant concentration where the initial straight line intersects the plateau in the graph.

All the material used for evaluation were extensively cleaned with water and organic solvents.

Surface tension measurements

The surface tension of water-octanol-surfactant systems were evaluated at double of the CMC of the surfactant. The drop volume method was used to calculate the surface tension in the system following the ISO standard 9101 with minor modifications.[1] In brief, a drop of

aqueous solution of surfactant was slowly formed in the tip of a needle (d=1.82 mm) and then submerged in octanol using a syringe connected to the Krüss DSA25. The drop formation was halted as close as possible the maximum volume at which the drop detaches from the needle. At this point, the drop was left to stabilize for 2 minutes and then the drop was detached from the needle by pushing a little more of aqueous solution. A photo of the drop leaving the needle was captured using the camera connected to the Krüss DSA25. The diameter of the drop was measured, and the surface tension was calculated using the following equation:

$$\gamma = \frac{V\Delta\rho g}{4\pi d f} \qquad \text{eq 1}$$

Where γ is the surface tension (mN/m), V is the drop volume (cm^3), $\Delta\rho$ is de difference of density between water and octanol (g/cm^3), g is the acceleration due to gravity (cm/s^2), d is the outer diameter of the needle (cm), and f is a correction factor calculated using the following equation:

$$f = \left(2\pi\left(0.1478 + 0{,}2789\frac{d}{2\sqrt[3]{V}}\right) - 0.1662\left(\frac{d}{2\sqrt[3]{V}}\right)^2\right)^{-1} \qquad \text{eq 2}$$

All the material used for evaluation were extensively cleaned with water and organic solvents.

Foaming tests

Foaming capacity was evaluated following the IS0 6964 with some modifications. In brief, 700 mL of surfactant solutions containing 0.2% surfactant were prepared using deionized water or a solution with 200 ppm $CaCl_2$. The solutions were prepared be mixing the components during 60 minutes at 50°C. After 60 minutes, 50 mL of solution were poured in a 1000-mL glass measuring cylinder and the measuring cylinder was placed in a water bath at 50°C. Then, 600 mL of solution were poured in a chromatography column. A stainless-steel needle was placed in the tip of the column and the column was placed over the glass measuring cylinder. 450-cm were measured between the solution in the measuring cylinder and the tip of the stainless-steel needle. After place the column in the proper height, the valve in the chromatography column was open and 450-mL of solution were poured from the column to the measuring cylinder. After pouring the solution, foam high was measured at 30 sec, 3 min, 5 min and 15 min.

All the material used for evaluation were extensively cleaned with water and organic solvents.

Inhibition of secretion B-galactosidase from E.Coli

The test was done following the standard operating protocol of the Toxi-Chromo test supplied by EBPI.A mother solution of each surfactant was prepared at 1000 ppm . The bacteria inoculum was mixed with a reaction mixture and nutrient supplied with the kit and the toxicant, further 15 dilutions of the surfactant were prepared in the well plate through subsequent dilutions .Then a chromogen supplied with the kit was added in each well and incubated at 37 C for 30 min. The OD was read at 600 nm using a plate reader. The %

inhibition was measured by the OD variation (which is associated a blue colour development). EC50 was measured by plotting and fitting the % inhibition and surfactant concentration.

Inhibition of Protozoa growth

The test was performed following the standard procedure of the Protoxkit supplied by microbiotests . A dilution series of toxicant was prepared by serial 1:2 dilution with distilled water. 2 ml of each solution is placed in a UV cuvette. An inoculum of Protozoa was diluted to reach an OD of 0.04 at 440 nm then 40 ul of inoculum was added along a solution of 40 ul of nutrients in each dilution vials. The initial OD was measured for each vial and then incubated at 30 °C for 24 h. Inhibition was measured by % in variation of initial and final OD. EC50 was determined as the concentration of surfactant to obtain 50 % inhibition through fitting of the curve % inhibition vs surfactant concentration.

Zein Test

Zein (ca. 2.00g $\pm$ 0.20g) was added to a centrifuge tube in addition to 40 cm^3 0.5% surfactant solution. Each tube was manually shaken to ensure thorough mixing of the surfactant and zein before being placed in an incubator at 40 °C for 24hrs. The tubes were then centrifuged for 5 minutes at 4000 rpm and the supernatant discarded. The remaining zein was then washed with water (3 x 30mL) and dried in an oven at 60 °C for 4 days. Zein tests were run in triplicate for each surfactant, with the average of these results being reported. % dissolved zein was calculated using the following equation.

$$\%\, dissolved = \left(\frac{(initial\ mass\ zein - final\ mass\ zein)}{initial\ mass\ zein}\right) \times 100 \qquad \text{eq 3}$$

Results and Discussion

Technoeconomic assessment

Table S3 Techno economic assessment results from the Aspen simulation

	from furoic acid	From furfural	LAB
Total cost of the feedstock ($/Kg)	2.65	2.08	0.61
Operating cost utility ($/Kg)	0.04	0.018	0.0065
Capital costs ($/Kg)	0.009	0.021	0.016
Minimum selling price ($/Kg)	2.7	2.12	0.64
Heating requirement (KJ/Kg)	826	155.0	55.18
Cooling requirement l/Kg	---	52	71
Waste water treatment (ml/Kg)	62		---
CO2 emissions (g/Kg)	48.3	9.89	3.2

Solubility study

Results of the solubility tests were reported as temperature to solubilize 20 % of the surfactant. Table S4 are reported the results of solubility tests for different surfactant concentrations. Results are expressed as temperature to achieve the solubilization of 20 % of the surfactants.

Table S4 Results of the solubility of different surfactants

	Temperature to achieve full solubilization (°C)
SDS	41-43
LAS	>50
SAF-8	<0
SAF-12	22-25
SAF-16	48
SAF-12/SDS (6:1)	10

Resistance to hard water

Further studies were conducted on the resistance of the surfactants to hard water. The results are reported in Table S5. LAS and SDS proved to have very low resistance to hard water, exhibiting extensive precipitation in many test conditions, which translates to a low stability score. On the other hand, SAF-8 and SAF-12 show very high solubility with the highest stability score, which means that these surfactants are highly resistant to calcium concentration. Since most of the additives used commercially are for chelating calcium atoms, these results are promising to solve problems related to the toxicity of these compounds and reducing the cost of the final formulation.

Table S5 Results of hard water stability tests

	Total Score	Mean Stability
SDS	21	2
LAS	50	3
SAF-8	75	5
SAF-12	75	5

Critical micelle concentration and surface tension water-octanol

Table S6 Calculated CMC for different surfactants

Surfactant	CMC (ppm)
SDS	2050
LAS	593
SAF-8	1332
SAF-12	521
SAF-16	60
SAF-12/SDS (6:1)	950<br>330

Table S7 Surface tension in pure and hard water(*500 ppm* $CaCl_2$)/octanol for SAF-8 and SAF-12 at double CMC concentration.

**Surfactant**	**Surface tension hard water (mN/cm)**	**Surface tension pure water (mN/cm)**
SAF-8 (2* CMC)	1.91±0.25	2.65±0.32
SAF-12 (2* CMC)	4.51±0.50	3.42±0.5
LAS	Precipitate obsered	4.48±0.75

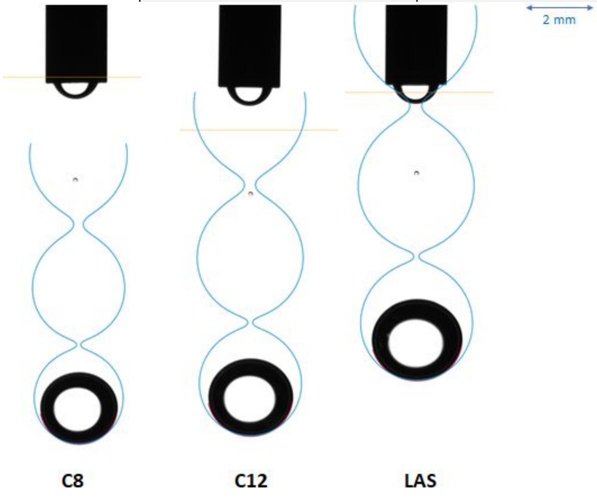

Figure S 1 Drop formation of a water/surfactant solution in octanol performed at a concentration of surfactant double of the CMC

Foam Test

Table S8 Foam test results evaluated after 5 min in distilled and hard water

	Distilled water (mm)	Hard water (mm)
LAS	9.25±	12.9±0.1
SDS	7.25±	4.25±0.4
SAF 8	2.5±	5±0.5
SAF 12	7.8±	10.35±0.5
SDS/SAF 12 1:6	9.9 ±	11.12±0.5

Evaluation Ecotoxicity

Table S9 Ecotoxicity test results

Surfactant	EC50 Inhibition B-galactosidase	EC50 Inhibition Protozoa growth
LAS	71.9±2.8 ppm	26.9± 4.8 ppm
SDS	123.7±5.2 ppm	85.71 ± 3.8 ppm
SAF 8	292±13 ppm	673 ±10 ppm
SAF 12	63.5±2.8 ppm	49.65±5 ppm
SDS/SAF 12 1:6	62.3±2.5 ppm	45± 10 ppm

Zein Test

Table S10 Zein test results

Surfactant	% Zein solubilized
LAS	24.6±1.85 %
SDS	9.75 ±0.78 %
SAF 8	10.4±0.98 %
SAF 12	15.3 ± 0.26 %
SDS/SAF 12 1:6	25 ± 0.8 %

NMR Spectra

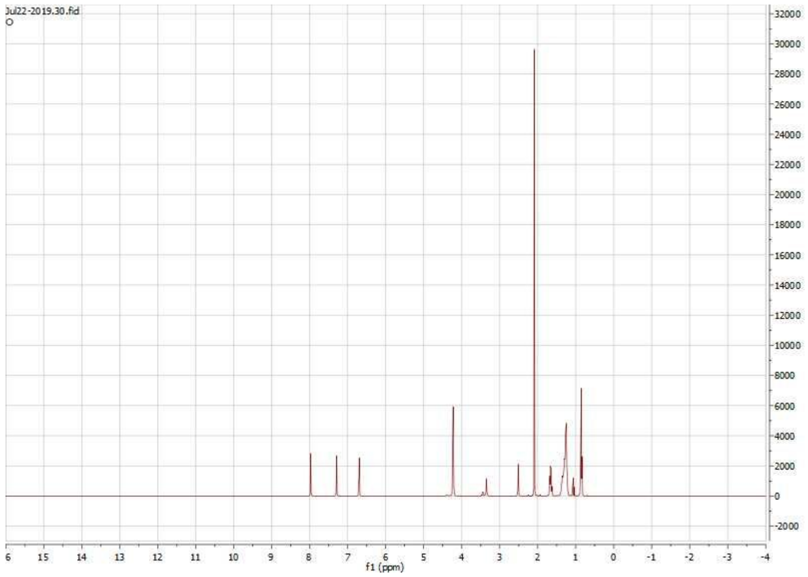

Figure S 2 H-NMR octyl furoate

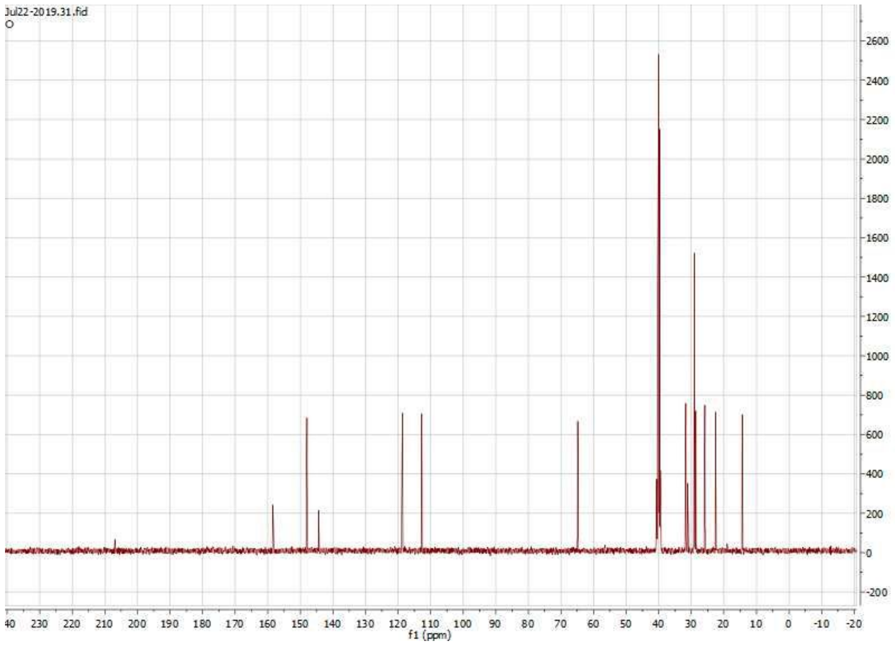

Figure S 3 C-NMR octyl furoate

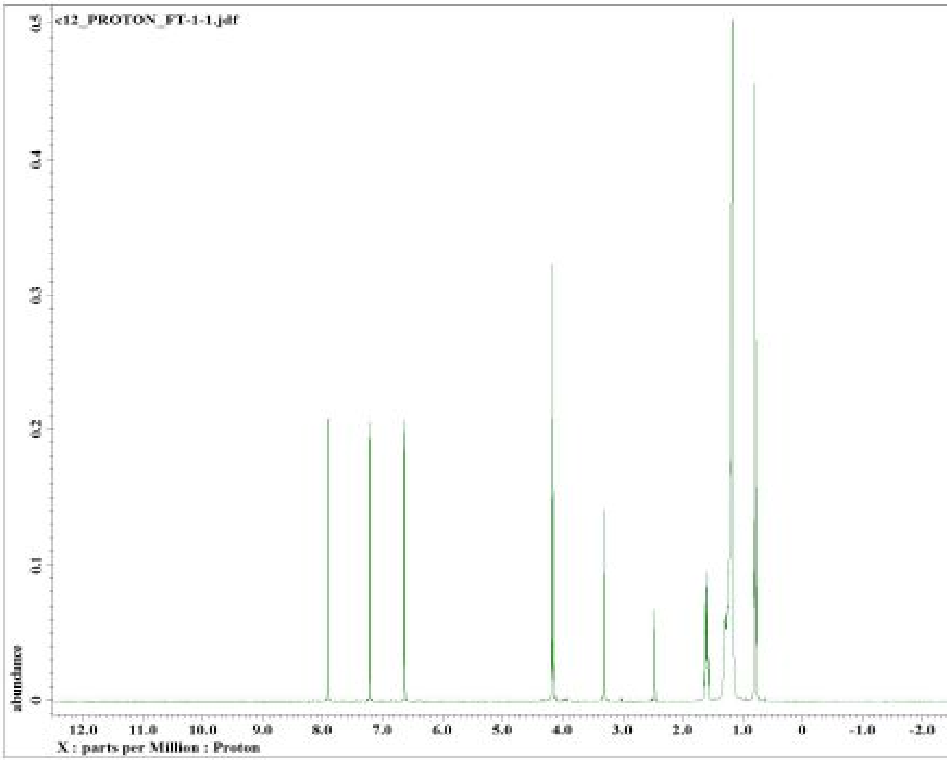

Figure S 4 H-NMR dodecyl furoate

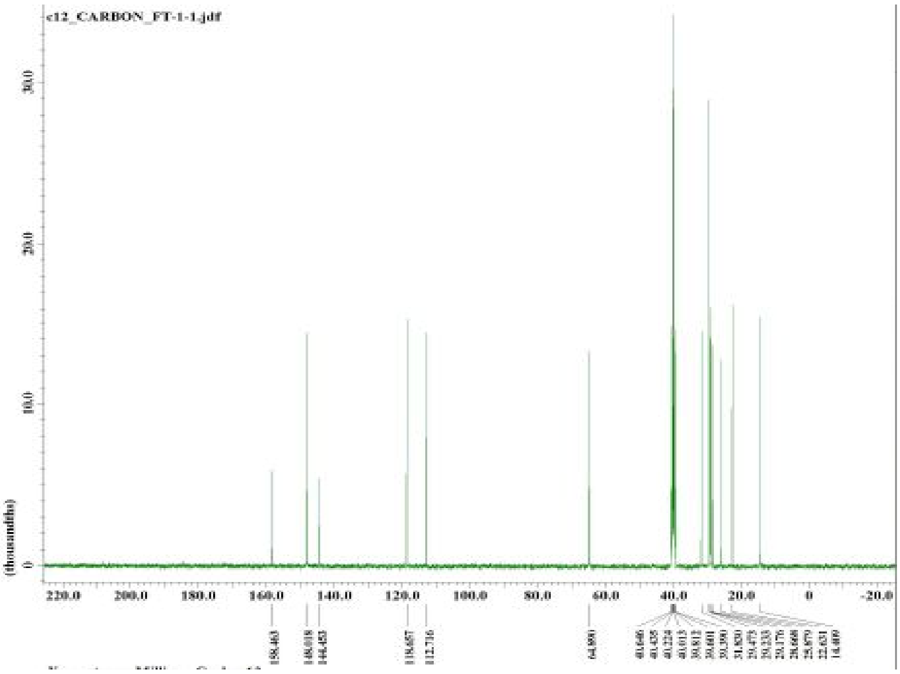

Figure S 5 C-NMR dodecyl furoate

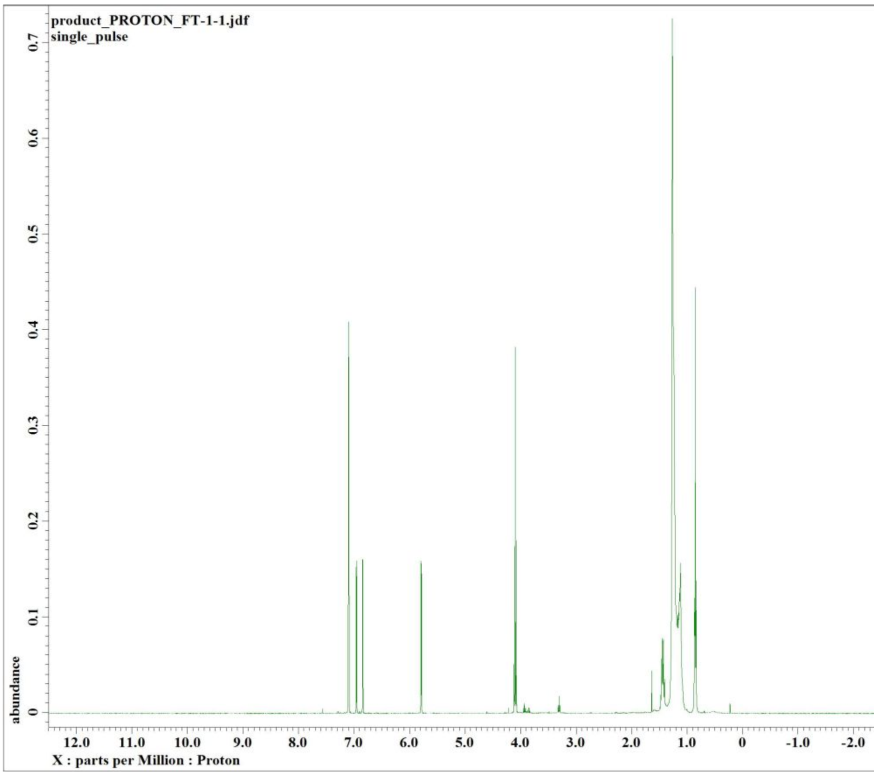

Figure S 6 H-NMR Hexadecyl Furoate

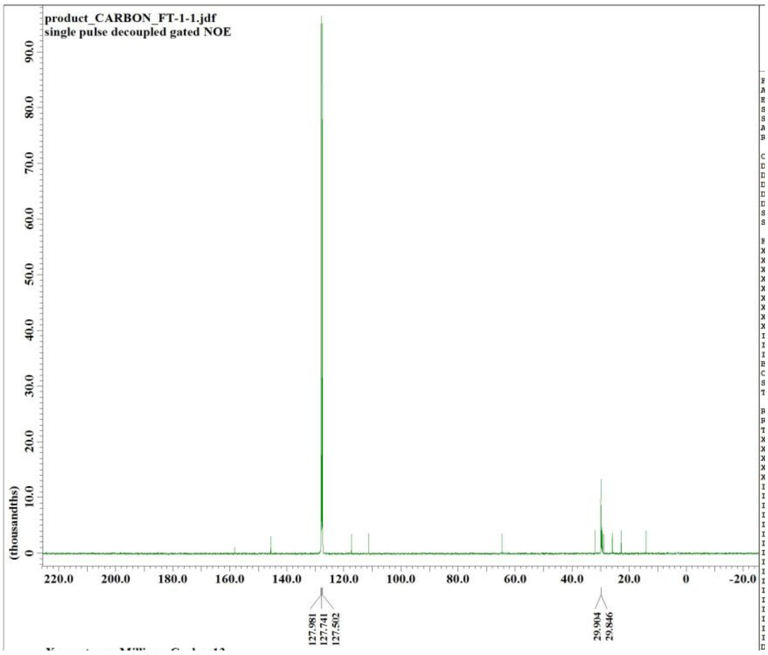

Figure S 7 C-NMR hexadecyl Furoate

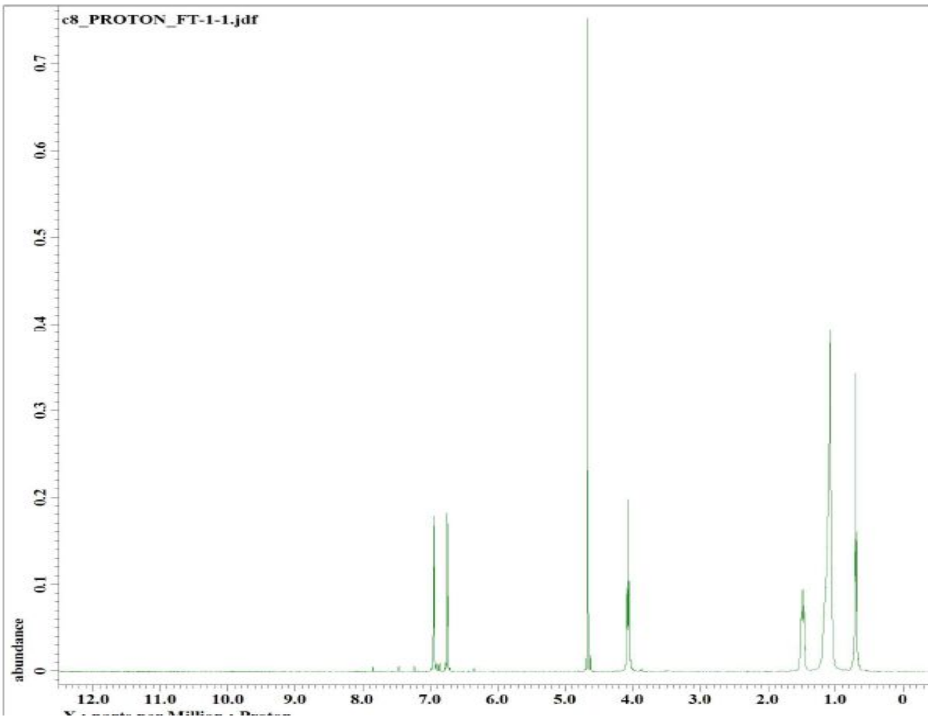

Figure S 8 H-NMR Sulphonated Octyl Furoate

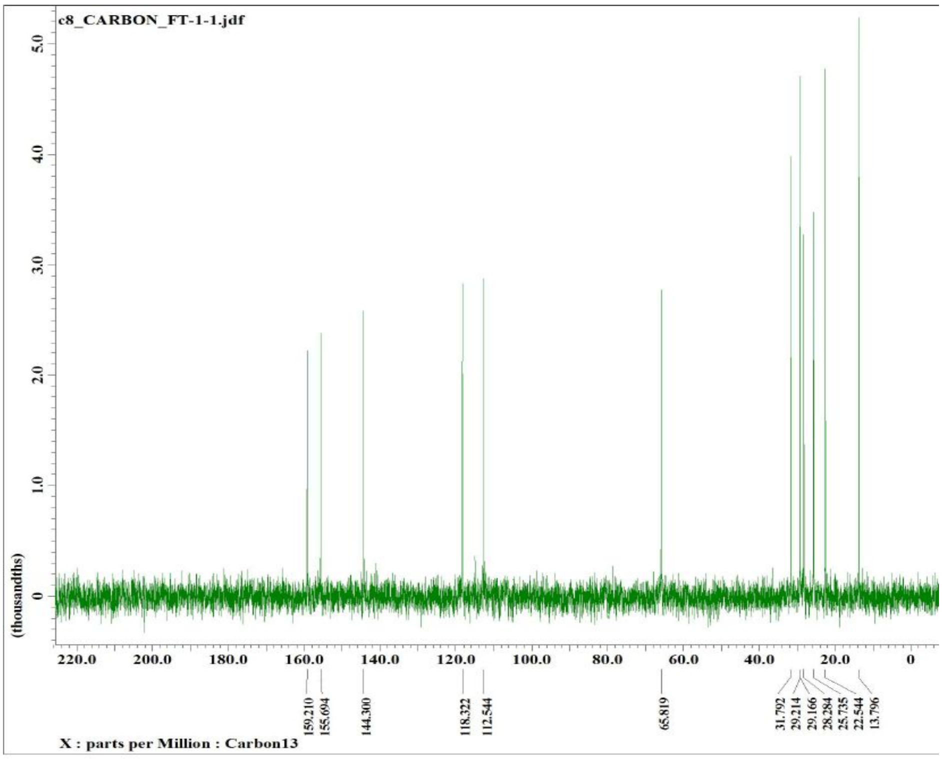

Figure S 9 C-NMR Sulphonated Octyl Furoate

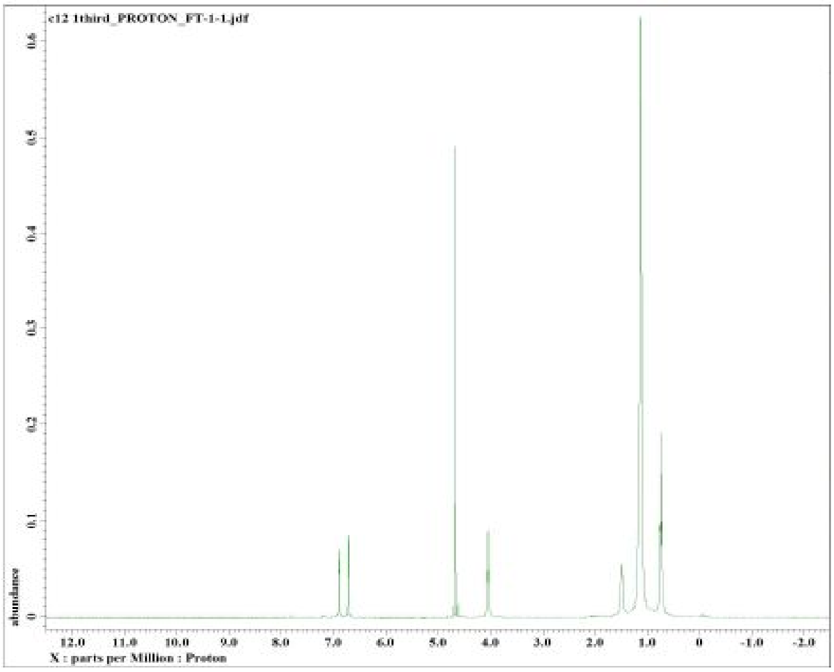

Figure S 10 H-NMR Sulphonated dodecyl furoate

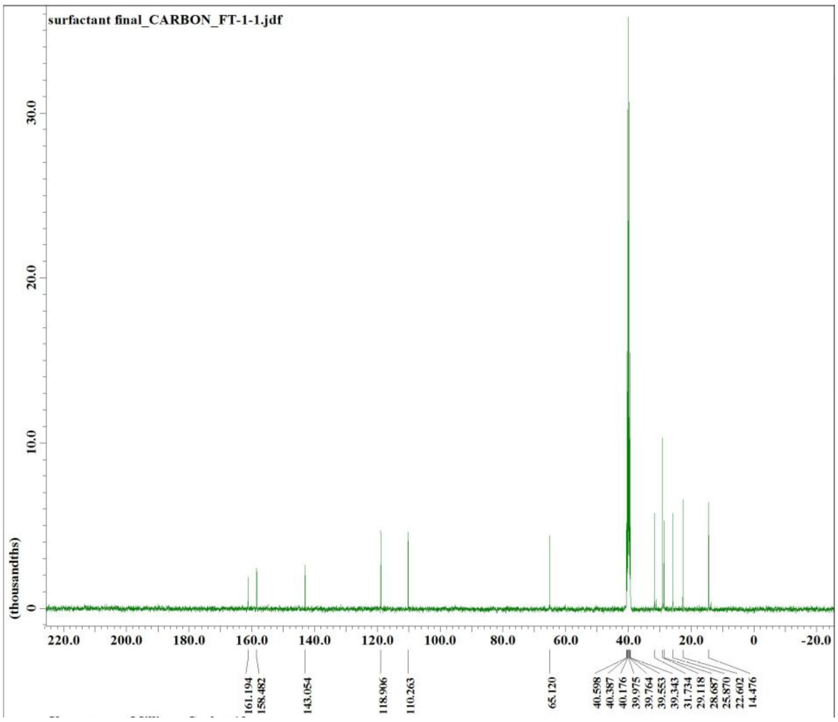

Figure S 11 C-NMR Sulphonated Dodecyl Furoate

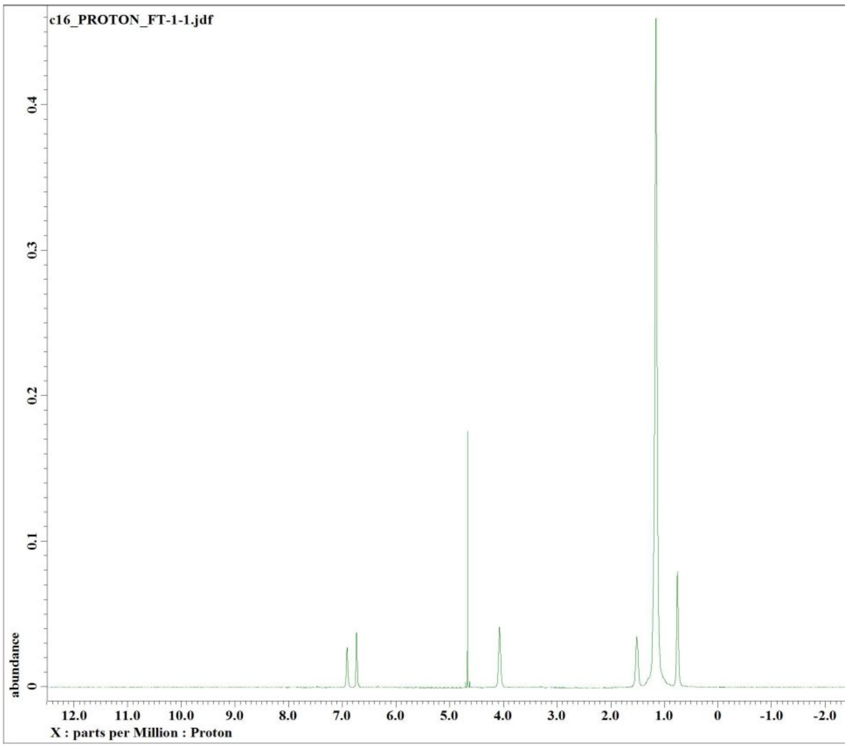

Figure S 12 H-NMR Sulphonated hexadecyl furoate

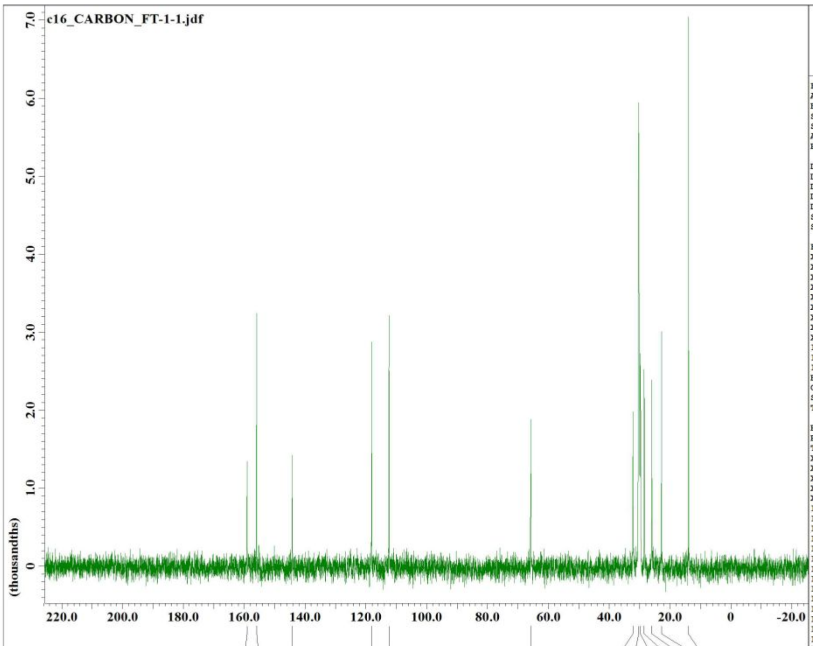

Figure S 13 C-NMR sulphonated hexadecyl furoate

Mass spectra

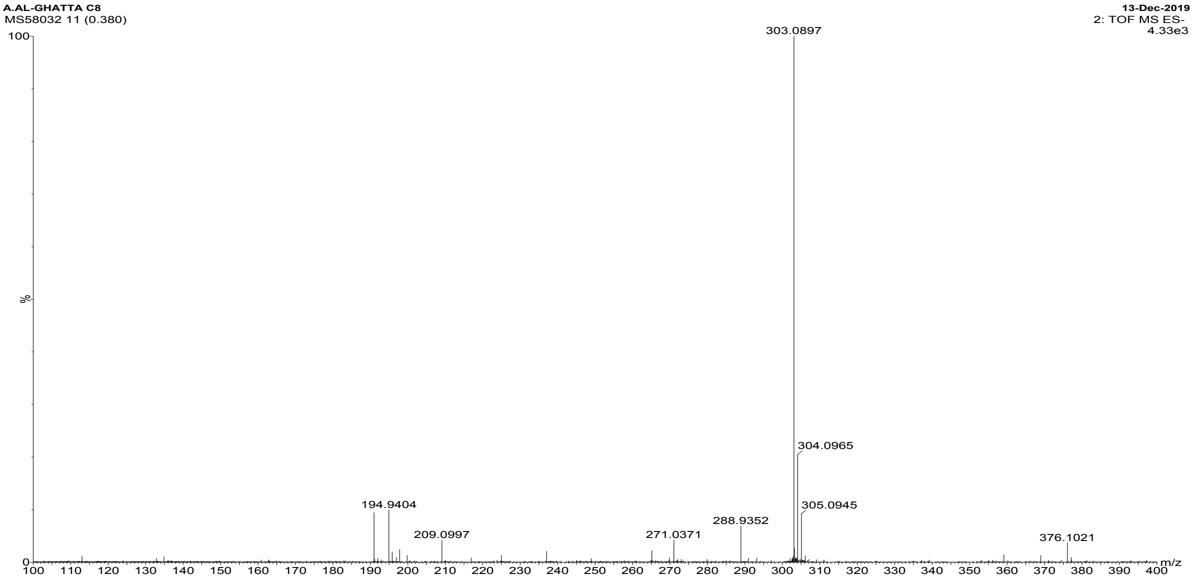

Figure S 14 Mass spectrum Sulfonated Octyl Furoate

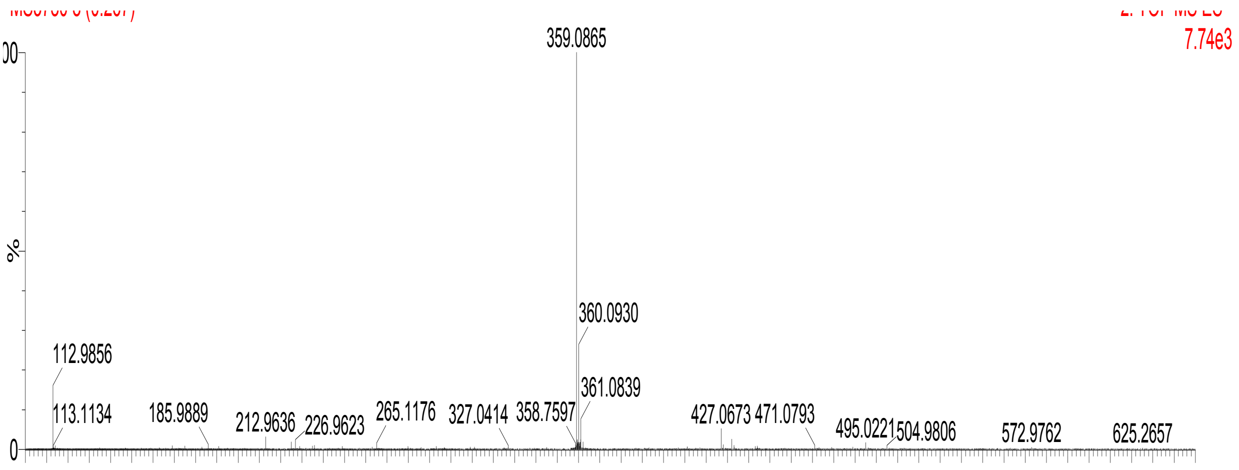

Figure S 15 Mass spectrum Sulfonated Dodecyl Furoate

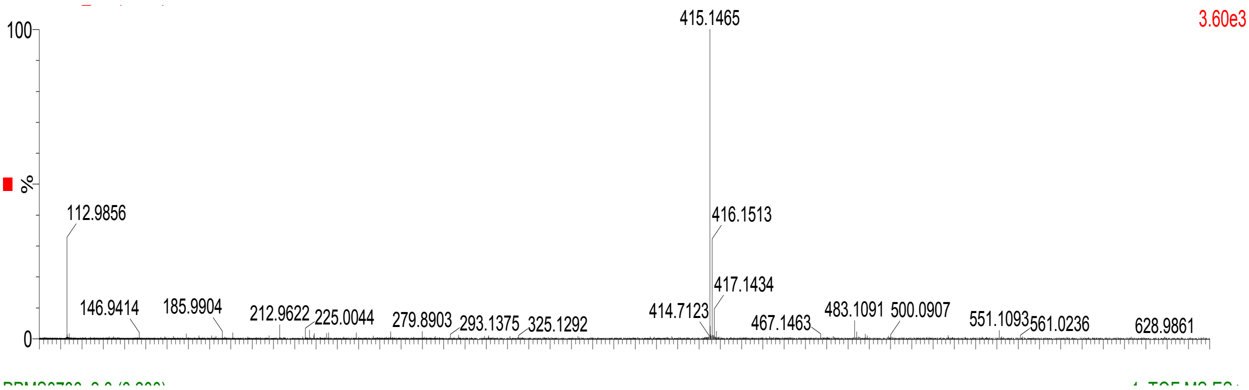

Figure S 16 Mass spectrum sulphonated hexadecyl furoate

FT-IR

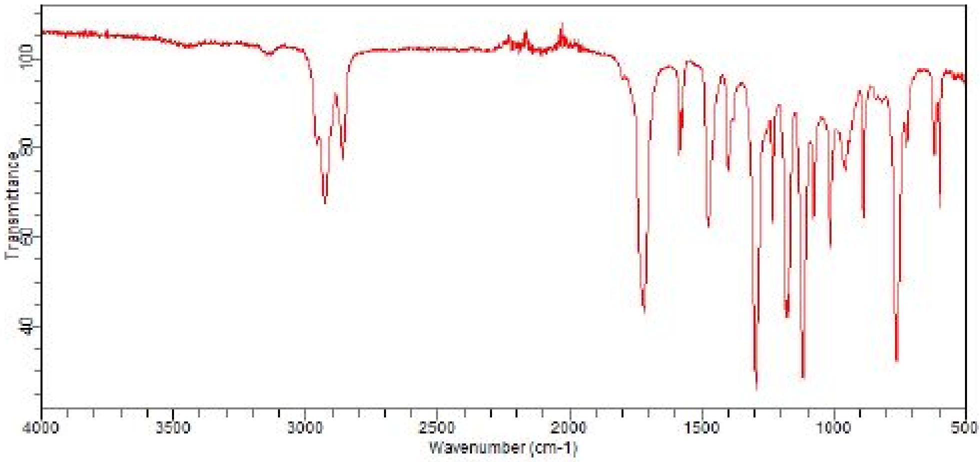

Figure S 17 FT-IR Octyl furoate

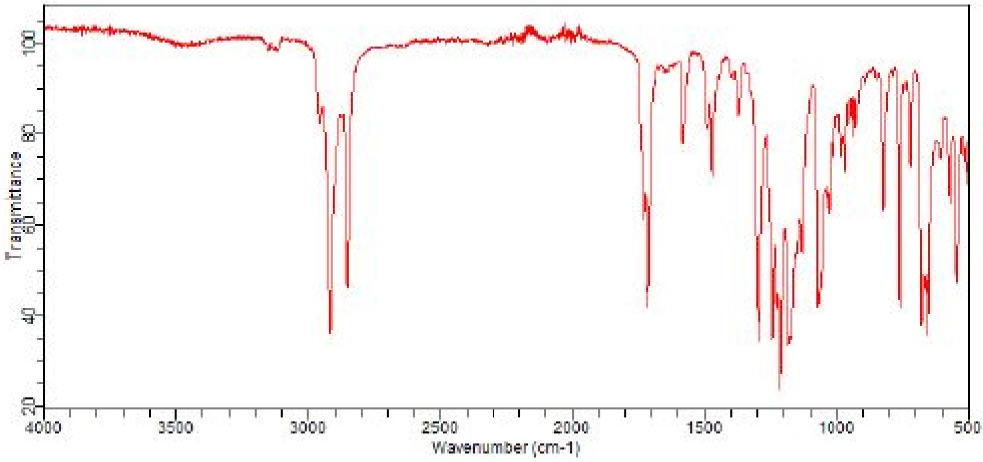

Figure S 18 FT-IR Sulphonated octyl furoate

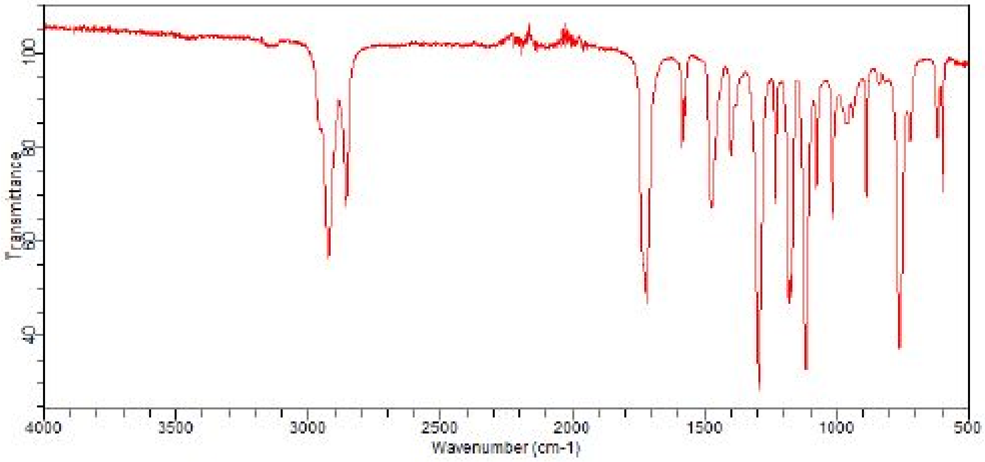

Figure S 19 FT-IR dodecyl furoate

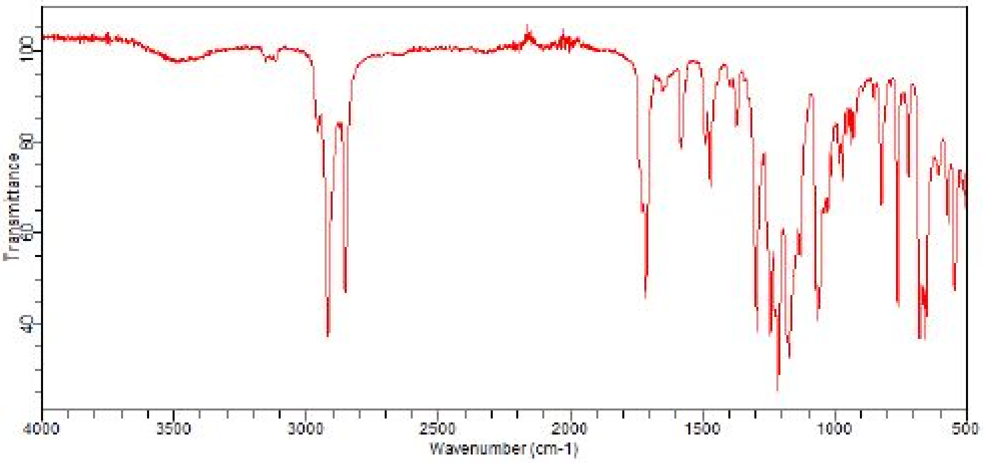

Figure S 20 FT-IR Sulphonated dodecyl furoate

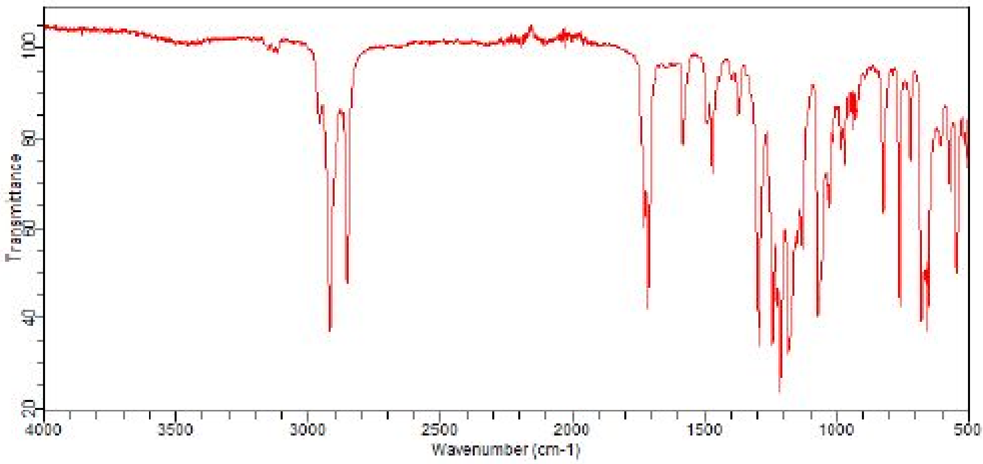

Figure S 21 FT-IR Hexadecyl furoate

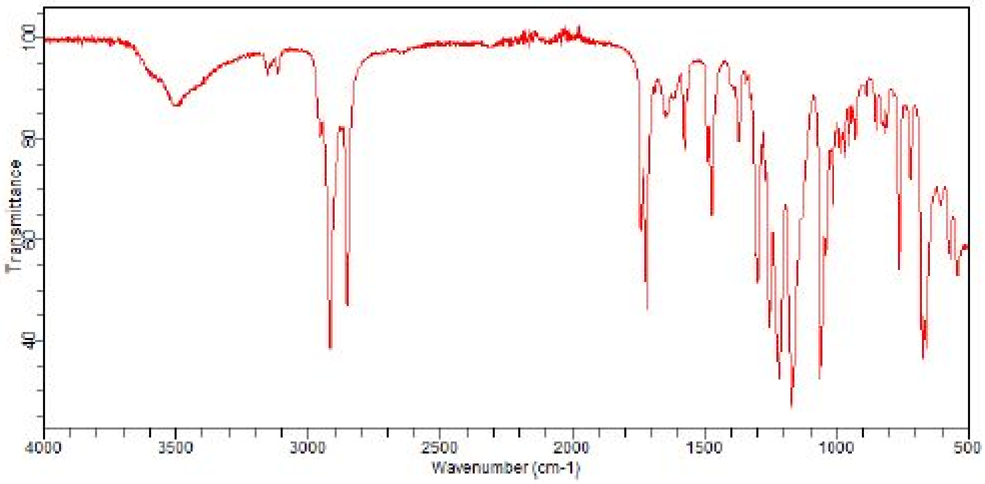

Figure S 22 FT-IR sulphonated hexadecyl furoate

CMC Measurement

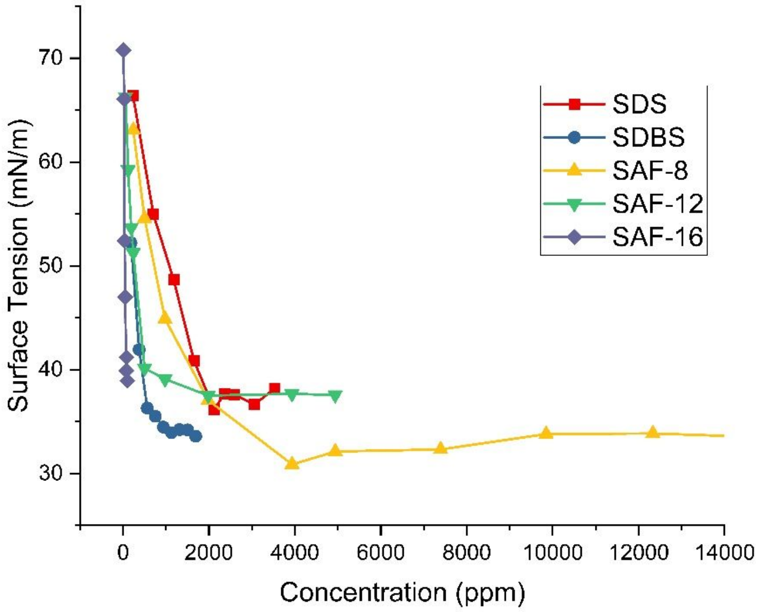

Figure S 23 Measurement of surface tension at different concentrations

Surface tension measurement

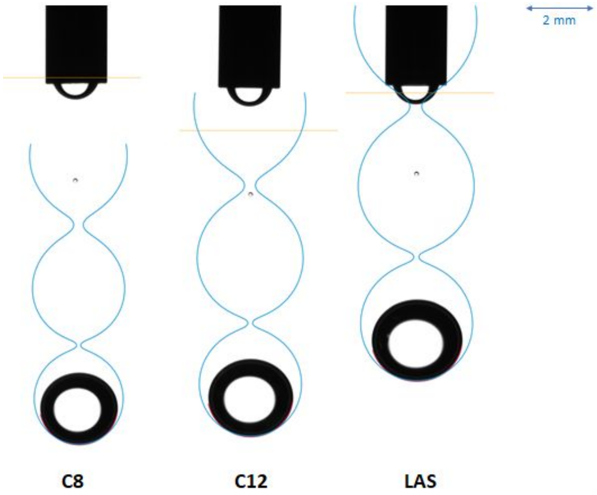

Figure S 24 Drop formation of a water/surfactant solution in octanol performed at a concentration of surfactant double of the CMC

References

1 Z. Q. Zhang and Y. H. Mori, *Ind. Eng. Chem. Res.*, 1993, **32**, 2950–2952.
